# Prevention and treatment of anthracycline-induced cardiotoxicity: a systematic review and network meta-analysis of randomized controlled trials

**DOI:** 10.1186/s40959-025-00360-3

**Published:** 2025-07-10

**Authors:** Siyu Li, Wenrui Li, Mengfei Cheng, Xiaoxiao Wang, Wanyi Chen

**Affiliations:** Department of Pharmacy, Chongqing University Cancer Hospital, Chongqing, China

**Keywords:** Anthracyclines, Cardiotoxicity, Randomized controlled trials, Network meta-analysis, Systematic review

## Abstract

**Background:**

Anthracyclines are cornerstone chemotherapeutics, but cardiotoxicity limits their use.

**Objective:**

This study aims to evaluate the efficacy of various drugs in preventing and treating anthracycline-induced cardiotoxicity (AIC).

**Methods:**

We conducted an extensive search across seven databases to identify randomized controlled trials (RCTs) pertinent to the prevention and treatment of AIC with medications. Subsequently, a Bayesian Model-based network meta-analysis was performed in the R 4.4.0.

**Results:**

A total of 128 RCTs involving 10,431 cancer patients treated with anthracyclines and 78 drug regimens were included in this study. The network meta-analysis results showed that, compared with patients who did not receive cardioprotective drugs, those treated with Calcium Dibutyryladenosine Cyclophosphate (Mean Difference [95% Credible Interval], 8.760 [0.5917, 16.92]), Carvedilol (4.024 [0.5372, 7.656]), Carvedilol + Candesartan (7.934 [3.159, 12.91]), Compound Salvia Miltiorrhiza + Levocarnitine (9.087 [0.9160, 17.25]), Dexrazoxane (5.066 [2.589, 7.540]), Dexrazoxane + Cinobufacini (11.61 [4.590, 18.70]), Dexrazoxane + Shenqi Fuzheng (13.05 [4.640, 21.40]), Nicorandil (14.24 [5.122, 23.31]), Qiliqiangxin (11.38 [2.826, 19.91]), and Xinmai Long (6.371 [1.735, 11.02]) experienced less decrease in LVEF after chemotherapy. The SUCRA ranking results indicated that the most effective treatment option for preserving LVEF was Nicorandil (SUCRA 91.76%).

**Conclusion:**

Apart from Dexrazoxane, Carvedilol, a β-blocker, also appears to show significant potential in preventing AIC. Furthermore, our results indicate that there is insufficient evidence to support the beneficial effects of statins, Sildenafil, Ivabradine, Levocarnitine, N-acetylcysteine, Glutathione, Coenzyme Q10, Vitamin E, and Vitamin C in preventing LVEF decline and exerting a positive effect on the prevention of AIC.

**Supplementary Information:**

The online version contains supplementary material available at 10.1186/s40959-025-00360-3.

## Key messages

• Anthracyclines are cornerstone chemotherapeutics, but cardiotoxicity limits their use.

• 128 RCTs/10,431 patients assess 78 drug regimens for AIC prevention/treatment in the network meta-analysis.

• Apart from Dexrazoxane, Carvedilol also appears to show significant potential in preventing AIC.

• Combining Dexrazoxane with certain Chinese patent medicines may enhance cardioprotection.

• Lack of valid evidence for ARBs, ACEIs, MRAs, statins, Sildenafil, etc., in AIC prevention.

**Supplementary Information:**

The online version contains supplementary material available at 10.1186/s40959-025-00360-3.

## Introduction

Despite the continuous introduction of novel anti-tumor drugs, anthracyclines remain a cornerstone in various combination chemotherapy regimens due to their notable features such as broad anti-tumor spectrum, favorable clinical efficacy, and effectiveness against hypoxic cells. Anthracyclines are widely used in the treatment of cancers including breast cancer, lymphoma, and leukemia [1]. A dose-limiting and potentially fatal adverse effect of anthracyclines is cardiotoxicity, which can occur at any stage of chemotherapy and manifests primarily as arrhythmias, pericardial effusion, myocardial ischemia, cardiomyopathy, and heart failure [2, 3]. Studies have reported that 6% of patients experience clinically symptomatic cardiac injury and 18% suffer from subclinical cardiac toxicity after anthracycline treatment [4]. Severe cardiotoxicity can significantly impair patients' cardiac function and health-related quality of life, posing substantial limitations on the clinical use of these drugs [5].

Currently, it has been demonstrated that the concomitant use of Dexrazoxane can effectively mitigate anthracycline-induced cardiotoxicity (AIC) [6], and Dexrazoxane has been exclusively recommended by the U.S. Food and Drug Administration (FDA) as a first-line agent for preventing AIC. However, studies have suggested that Dexrazoxane may increase the risk of second primary malignancies in pediatric cancer patients [7–9]. Besides Dexrazoxane, recent studies have successively identified that statins [10, 11], β-blockers [12, 13], renin–angiotensin–aldosterone system (RAAS) inhibitors [14, 15] (including angiotensin-converting enzyme inhibitors [ACEIs], angiotensin receptor blockers [ARBs], and mineralocorticoid receptor antagonists [MRAs]), as well as some Chinese patent medicines [16], may play a positive role in the primary prevention of AIC.

To date, there has been a lack of large-scale randomized controlled trials (RCTs) conducting head-to-head comparisons of these different medications. This systematic review and network meta-analysis of RCTs aims to evaluate the currently reported drugs for the prevention and treatment of AIC, further update and integrate the latest evidence, and support clinical decision-making.

## Method

This study is reported in accordance with the PRISMA statement for systematic reviews [17] and the PRISMA extension for network meta-analysis (PRISMA-NMA) [18], and has been successfully registered on PROSPERO (registration number CRD 42024552106). Patients and the public were not involved in the design, conduct, reporting, and dissemination plans of our study.

### Eligibility criteria

We established the eligibility criteria for the literature based on the PICOS (Population, Intervention, Comparator, Outcome, Study design) principle.

#### Inclusion criteria

1. Population (P): Cancer patients who are receiving or anticipated to receive anthracycline-based chemotherapy;
2. Intervention (I): Chemical drugs or Chinese patent medicines used for the prevention or treatment of AIC;
3. Comparator (C): Placebo, no intervention, or active drugs;
4. Outcome (O): Indicators related to cardiac injury, such as left ventricular ejection fraction (LVEF), electrocardiogram, and myocardial enzymes. During medication or follow-up, the occurrence of arrhythmias (atrial fibrillation, premature beats, tachycardia, bradycardia), low QRS voltage, ST-T abnormalities, prolonged Q-T interval, and conduction blocks are defined as electrocardiographic abnormalities in patients. Additionally, patients who exhibit one of the following: ① LVEF abnormalities, myocardial enzyme abnormalities, or electrocardiographic abnormalities, accompanied by clinical manifestations of cardiac dysfunction such as shortness of breath, fatigue, and dyspnea; ② patients who experience AIC classified as Grade II or higher according to the World Health Organization (WHO) Recommendations for Grading of Acute and Subacute Toxicity of Cancer Treatment [19];
5. Study design (S): RCTs.

#### Exclusion criteria

1. Patients concurrently receiving or anticipating other potentially cardiotoxic agents;
2. Reviews or editorials;
3. Conference abstracts, letters, study proposals, etc., that do not report detailed treatments and outcomes information;
4. Duplicate publications;
5. The full text is unavailable;
6. Non-Chinese and non-English languages.

### Search strategy and data sources

A comprehensive search was conducted in the Medline (Ovid), Embase (Ovid), Cochrane Central Register of Controlled Trials, Web of Science, CNKI, WanFang Data, and VIP databases to collect RCTs on the prevention and treatment of AIC with medications. The search covered the period from the inception of each database up to June 2024. Besides, the reference lists of included studies and relevant reviews were traced as supplementary searches. The search strategies combined subject headings and free-text terms, and were adjusted slightly for each database. The search terms included: anthracycline, daunorubicin, doxorubicin, epirubicin, pirarubicin, aclarubicin, idarubicin, valrubicin, mitoxantrone; cardiotoxicity, arrhythmia, myocardial ischemia, cardiomyopathy, cardiac failure, heart failure, adverse cardiac event, adverse cardiac reaction, adverse cardiac effect; treatment, prevention, management, etc. No language restrictions were applied during the search process. The detailed search strategy is provided in the Supplementary file 1.

### Study selection

Before formally screening eligible studies, a preliminary test of the selection process was conducted. Two researchers independently selected potentially eligible studies based on the titles and abstracts, and then confirmed the final inclusion through full-text articles. Inconsistencies were resolved through negotiation between the two researchers or determined by a third one.

### Data extraction

Two trained researchers independently extracted data using a standardized form and cross-checked the extracted data. Any disputes were resolved through discussion between the two researchers or determined by a third one. The extracted data included: ① basic information of the literature (first author, title, publication year, etc.); ② study design (study setting, follow-up duration, etc.); ③ basic characteristics of patients (age, gender, cancer type, chemotherapy regimens, etc.); ④ treatments (drug name, dosage form, administration regimen, etc.); ⑤ outcome data (LVEF, electrocardiogram, and other indicators related to cardiac function). This study prioritized the intention-to-treat (ITT) results or adjusted ITT results reported in the studies over per-protocol analysis results. For studies with non-combinable different dose groups of the same treatment, the results of the highest dose data were extracted. For studies with two or more follow-up time points, the results of the longest follow-up time point data were extracted.

### Bias risk assessment

Two researchers independently assessed the risk of bias in the studies using the Cochrane Risk of Bias Tool 2.0 (ROB 2.0) [20]. The ROB 2.0 provides a series of signaling questions (with answer options of "yes," "probably yes," "probably no," "no," and "no information") to help assess the risk of bias in the following six domains:"bias arising from the randomization process," "bias due to deviations from intended interventions," "bias due to missing outcome data," "blinding of outcome assessment," and "bias in selection of the reported result"(with risk levels of low, some concerns, and high). The overall risk of bias in the RCTs was assessed with reference to the Cochrane Handbook [20]. A trial was considered to have a low overall risk of bias when all domains were judged as low risk, and a high overall risk of bias when the trial was judged to be at high risk of bias in at least one domain (Table 1).

**Table 1:** Overall risk of bias

Low risk of bias	Some concerns	High risk of bias
The trial is judged to be at low risk of bias for all domains for this result	The trial is judged to raise some concerns in at least one domain for this result, but not to be at high risk of bias for any domain	The trial is judged to be at high risk of bias in at least one domain for this result Or The trial is judged to have some concerns for all domains in a way that substantially lowers confidence in the result

### Data synthesis and statistical analysis

This study conducted a network meta-analysis using a random-effects model within a Bayesian framework. Each node represented a treatment, with the placebo/no treatment group serving as the common control group. For dichotomous outcomes, the odds ratio (OR) and corresponding 95% credible interval (95% CrI) were used to summarize the effects of different treatments. For continuous outcomes, the mean difference (MD) and corresponding 95% CrI were used. A two-sided *p*-value < 0.05 was considered statistically significant. Besides, for treatment groups using multiple drugs, this study regarded them as a combined regimen and created separate nodes.

The gemtc and ggplot2 packages in R version 4.4.0 were used for network meta-analysis and plotting graphs. In the network relationship graph, the size of nodes represented the number of patients, and the width of each line indicated the number of direct comparisons studies between the two treatments. Based on the ranking probability matrix table generated by the R, cumulative ranking probability curves were plotted, and the surface under the cumulative ranking curve (SUCRA) was calculated. A higher SUCRA value generally indicated a higher-ranking probability for the treatment.

Furthermore, our study conducted sensitivity analyses using different methods (fixed-effects and random-effects models) for combining effect sizes to assess the stability of the results, and we evaluated the agreement of direct and indirect evidence in the studied treatment networks using node-splitting analysis.

## Results

### Literature search and screening

**Fig. 1:**
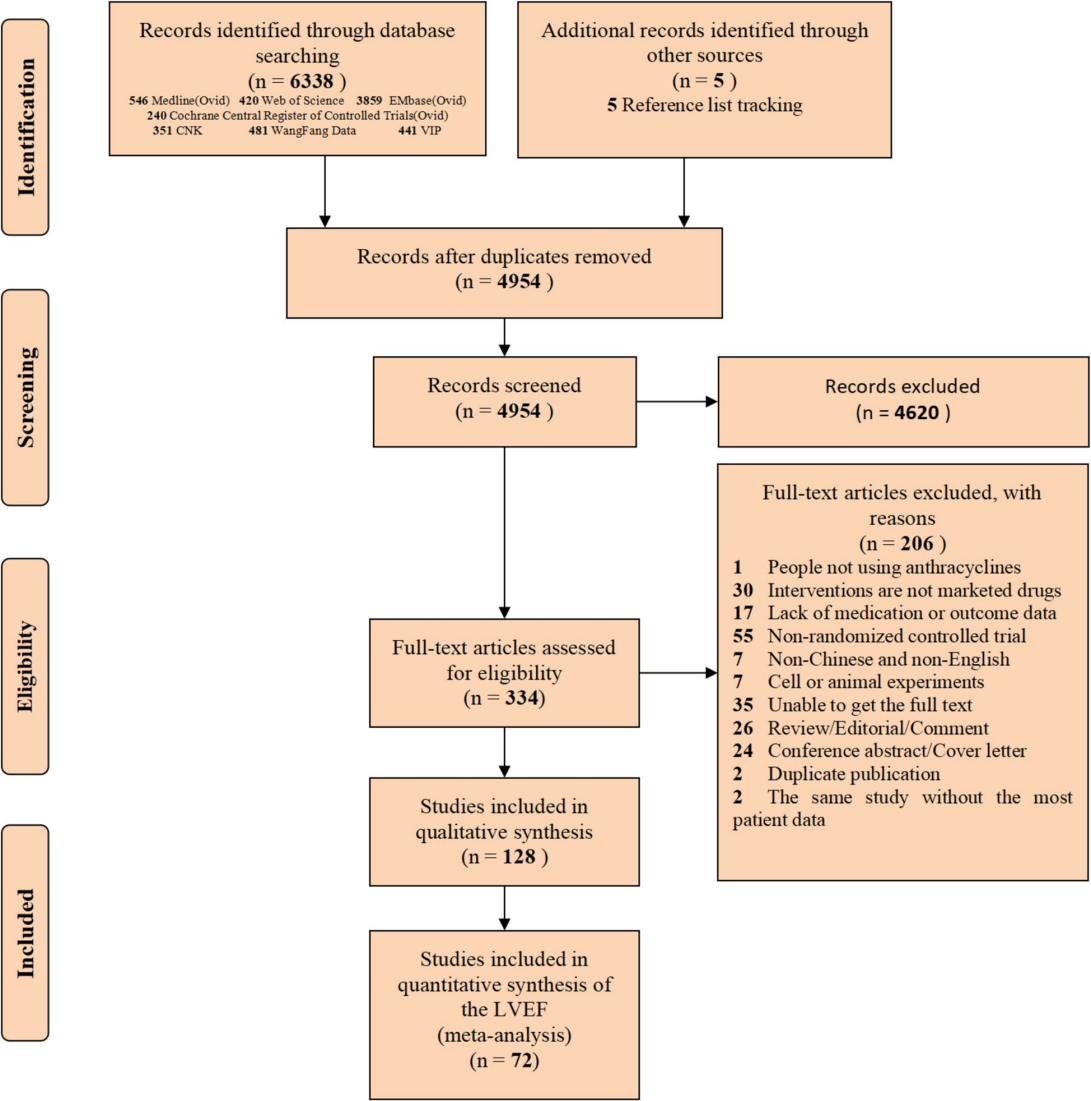
Research screening process

A total of 6338 articles were searched from various databases, and 5 articles were obtained from reviews and the reference lists of included studies. After removing duplicates, 4954 articles were initially screened by browsing the titles and abstracts. Excluding 4620 irrelevant articles, 334 articles underwent full-text screening, ultimately resulting in the inclusion of 128 studies [10, 21–147] with 10,431 patients (Fig. 1).

### Characteristics of studies

The 128 RCTs, published between 1996 and 2024, reported 78 drug regimens (41 chemical drugs, 10 combined chemical and Chinese patent medicines, and 27 Chinese patent medicines). In these RCTs, 3841 patients were randomly assigned to the placebo or no treatment group, while 6590 patients were randomly assigned to cardioprotective drug groups. Only 9 RCTs (7.0%) recruited patients aged ≤ 18 years, and the average age of patients in over half of the studies was between 40 and 60 years. The duration of follow-up ranged from 1 day to 5 years, with a median follow-up time of 4 to 6 months in most studies. In terms of the types of anthracyclines used, 50 RCTs (39.1%) involved doxorubicin, and 29 studies (22.7%) involved epirubicin. The top five countries with the highest number of studies were China (104 RCTs, 81.3%), Iran (3 RCTs, 2.3%), Canada (2 RCTs, 1.6%), Korea (2 RCTs, 1.6%), and Turkey (2 studies, 1.6%) (Supplementary file 2).

### Risk of bias assessment

Twelve RCTs (9.4%) were assessed as high risk of bias, 108 RCTs (84.4%) were considered as having "some concerns", and 8 RCTs (6.3%) were assessed as low risk of bias. In the domain of "bias arising from the randomization process", 10 RCTs (7.8%) were assessed as high risk, 48 RCTs (37.5%) were assessed as low risk, and 70 RCTs (54.7%) were deemed to have "some concerns" due to inadequate reporting of the randomization process and lack of details on allocation concealment. In the domain of "bias due to deviations from intended interventions," most studies were assessed as having "some concerns"(114 RCTs [89.1%]). Regarding "blinding of outcome assessment", 23 RCTs (18.0%) were assessed as low risk, while 105 RCTs (82.0%) were considered as having "some concerns" due to insufficient reporting of relevant information. For "bias in selection of the reported result" and "bias due to missing outcome data", 123 RCTs (96.1%) and 125 RCTs (97.7%), respectively, were assessed as low risk of bias (Supplementary file 3).

### Prevention of anthracycline-induced cardiotoxicity

Assessments of heterogeneity and consistency for all outcomes are reported in the Supplementary file 4.

#### Left ventricular ejection fraction

Seventy-two RCTs [10, 21–31, 33–41, 45–49, 51–54, 57–59, 61–64, 67, 68, 71, 75, 76, 80, 85, 86, 88, 89, 93, 95–98, 101, 104, 111, 112, 118–120, 123, 127–131, 133, 134, 136, 139, 140, 144, 146] reporting LVEF were included in the network meta-analysis. These studies reported 46 drug regimens (25 chemical drugs, 4 combined chemical and Chinese patent medicines, and 17 Chinese patent medicines) and involved 5928 patients using anthracyclines (2346 patients were randomly assigned to the placebo or no treatment group, while 3582 patients were randomly assigned to the cardioprotective drug groups).

**Fig. 2:**
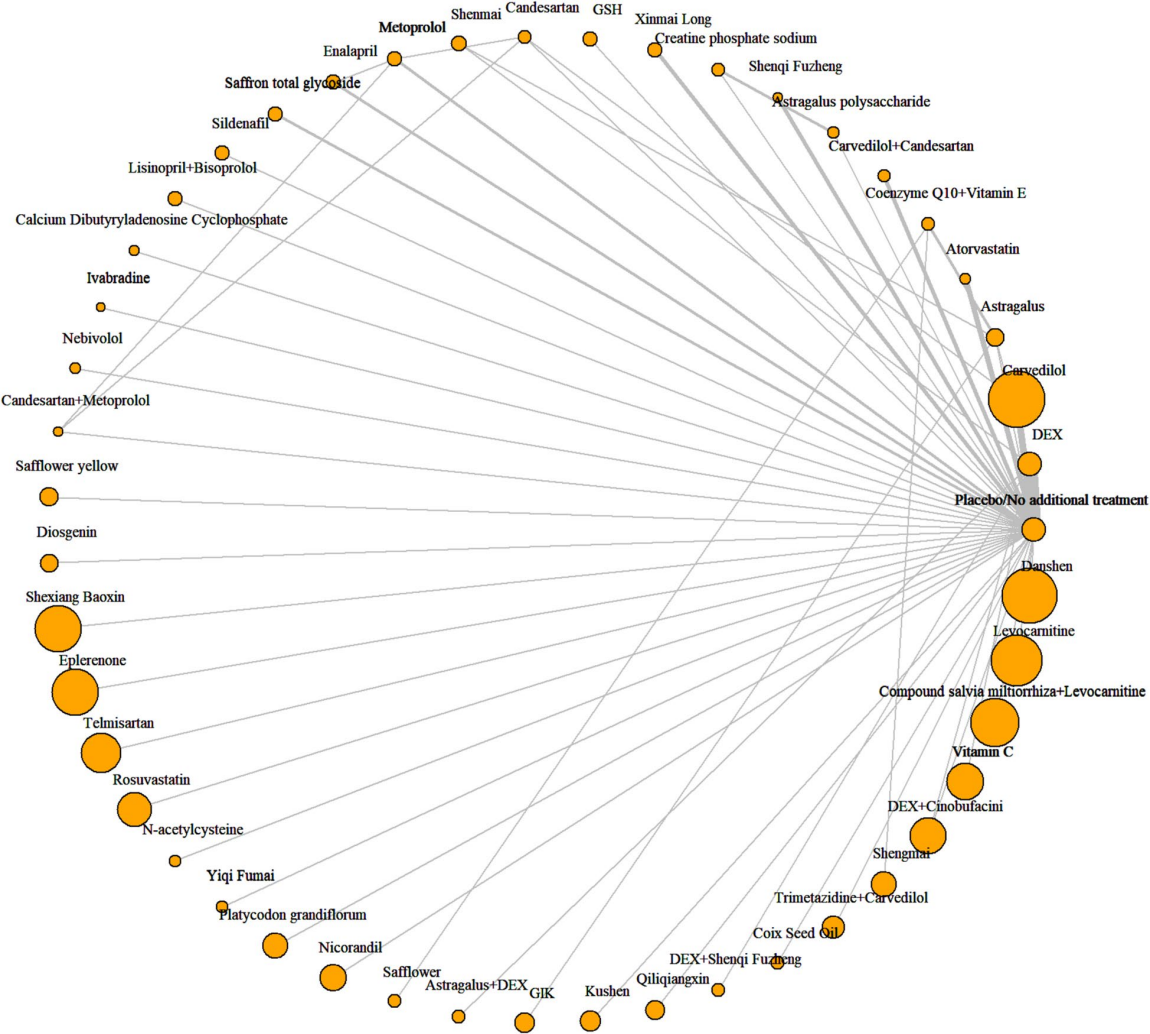
Network of LVEF. Note: DEX: Dexrazoxane; GSH: Reduced glutathione; GIK: Glucose-insulin-potassium. Astragalus, Astragalus polysaccharide, Coix Seed Oil, Compound salvia miltiorrhiza, Cinobufacini, Danshen, Diosgenin, Kushen, Platycodon grandiflorum, Qiliqiangxin, Safflower, Safflower yellow, Saffron total glycoside, Shengmai, Shenmai, Shenqi Fuzheng, Shexiang Baoxin, Xinmai Long, and Yiqi Fumai are Chinesel Patent Medicines, and most are injections (Diosgenin, Platycodon grandiflorum, Qiliqiangxin, Saffron total glycoside, and Shexiang Baoxin are solid oral dosage forms, including tablets, pills, capsules, and granules)

All treatments, except for Shenmai, Safflower, Glucose-Insulin-Potassium (GIK), Dexrazoxane (DEX) + Shenqi Fuzheng, Shengmai, Vitamin C, and Coenzyme Q10 + Vitamin E, had at least one placebo-controlled trial or no-treatment control trial (Fig. 2).

**Fig. 3:**
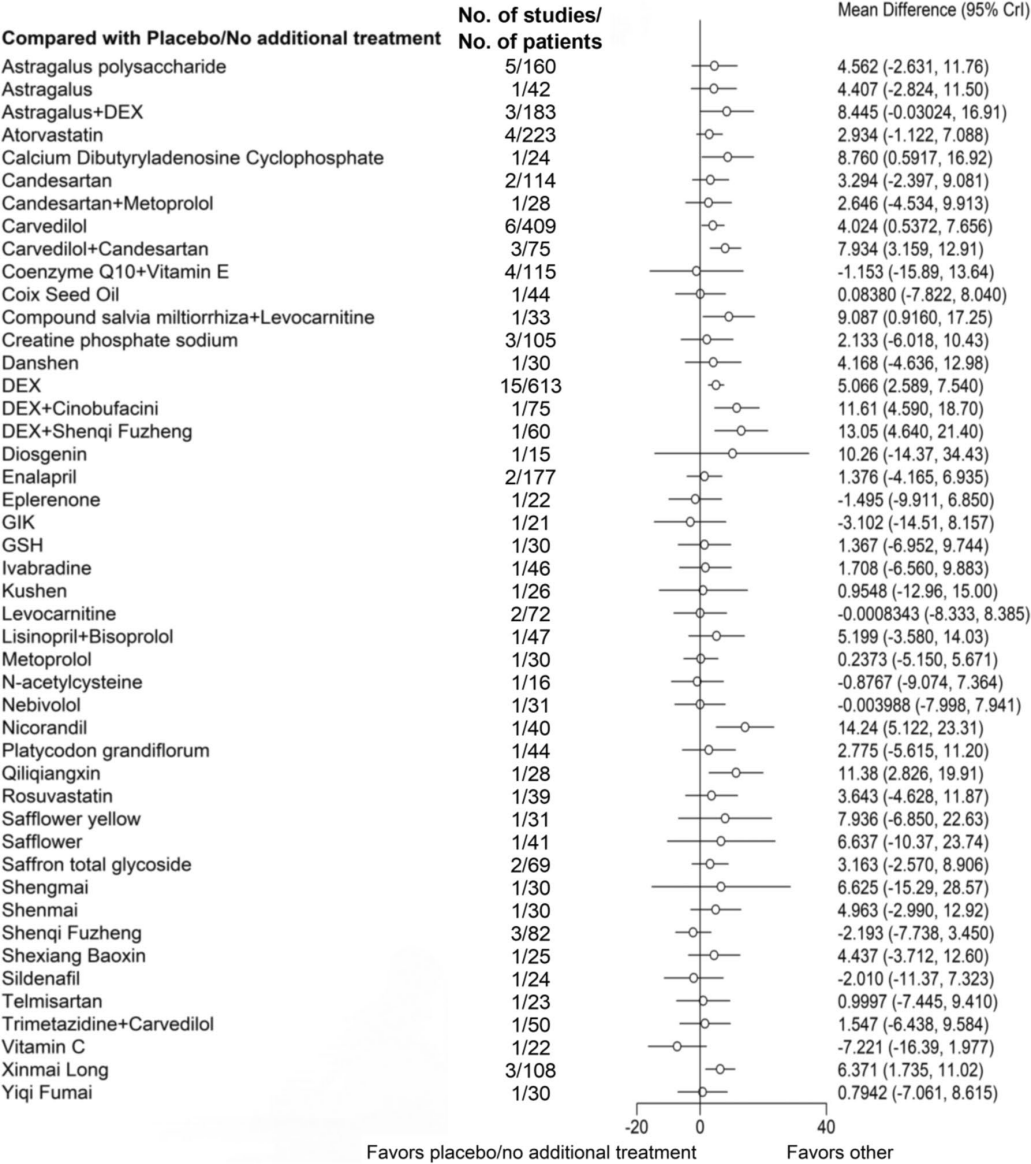
Network forest plot of LVEF. Note: DEX: Dexrazoxane; GSH: Glutathione; GIK: Glucose-insulin-potassium. Astragalus, Astragalus polysaccharide, Coix Seed Oil, Compound salvia miltiorrhiza, Cinobufacini, Danshen, Diosgenin, Kushen, Platycodon grandiflorum, Qiliqiangxin, Safflower, Safflower yellow, Saffron total glycoside, Shengmai, Shenmai, Shenqi Fuzheng, Shexiang Baoxin, Xinmai Long, and Yiqi Fumai are Chinese Patent Medicines, and most are injections (Diosgenin, Platycodon grandiflorum, Qiliqiangxin, Saffron total glycoside, and Shexiang Baoxin are solid oral dosage forms, including tablets, pills, capsules, and granules)

In terms of preventing LVEF decline after anthracyclines use, the following treatments were more effective than placebo/no treatment: Calcium Dibutyryladenosine Cyclophosphate (Mean Difference [95% CrI], 8.760 [0.5917, 16.92]), Carvedilol (4.024 [0.5372, 7.656]), Carvedilol + Candesartan (7.934 [3.159, 12.91]), Compound Salvia Miltiorrhiza + Levocarnitine (9.087 [0.9160, 17.25]), DEX (5.066 [2.589, 7.540]), DEX + Cinobufacini (11.61 [4.590, 18.70]), DEX + Shenqi Fuzheng (13.05 [4.640, 21.40]), Nicorandil (14.24 [5.122, 23.31]), Qiliqiangxin (11.38 [2.826, 19.91]), and Xinmai Long (6.371 [1.735, 11.02]) (Fig. 3).

Based on the cumulative probability plot and SUCRA rankings, the highest-ranked treatment is Nicorandil (SUCRA, 91.76%), followed by DEX + Shenqi Fuzheng (90.05%), and DEX + Cinobufacini (87.54%). The complete cumulative probability plot and SUCRA are provided in the Supplementary file 6.

#### Electrocardiographic abnormalities

A total of 70 RCTs [25, 29, 33, 39, 47, 49–51, 53–57, 62, 64, 66–69, 71, 73, 75, 77–81, 83, 85, 88, 89, 91, 93, 95, 97–101, 103, 105, 106, 111–117, 119, 123–134, 136, 137, 139–141, 144, 145, 147] reporting ECG abnormalities in patients were included in the network meta-analysis. These studies evaluated 43 drug regimens (21 chemical drugs, 6 combined chemical and Chinese patent medicines, and 16 Chinese patent medicines) involving 5,504 patients who received anthracyclines (2,061 patients were randomly assigned to the placebo or no treatment group, while 3,443 patients were randomly assigned to the cardioprotective drug groups. See Supplementary file 5 for the network relationship figure).

**Fig. 4:**
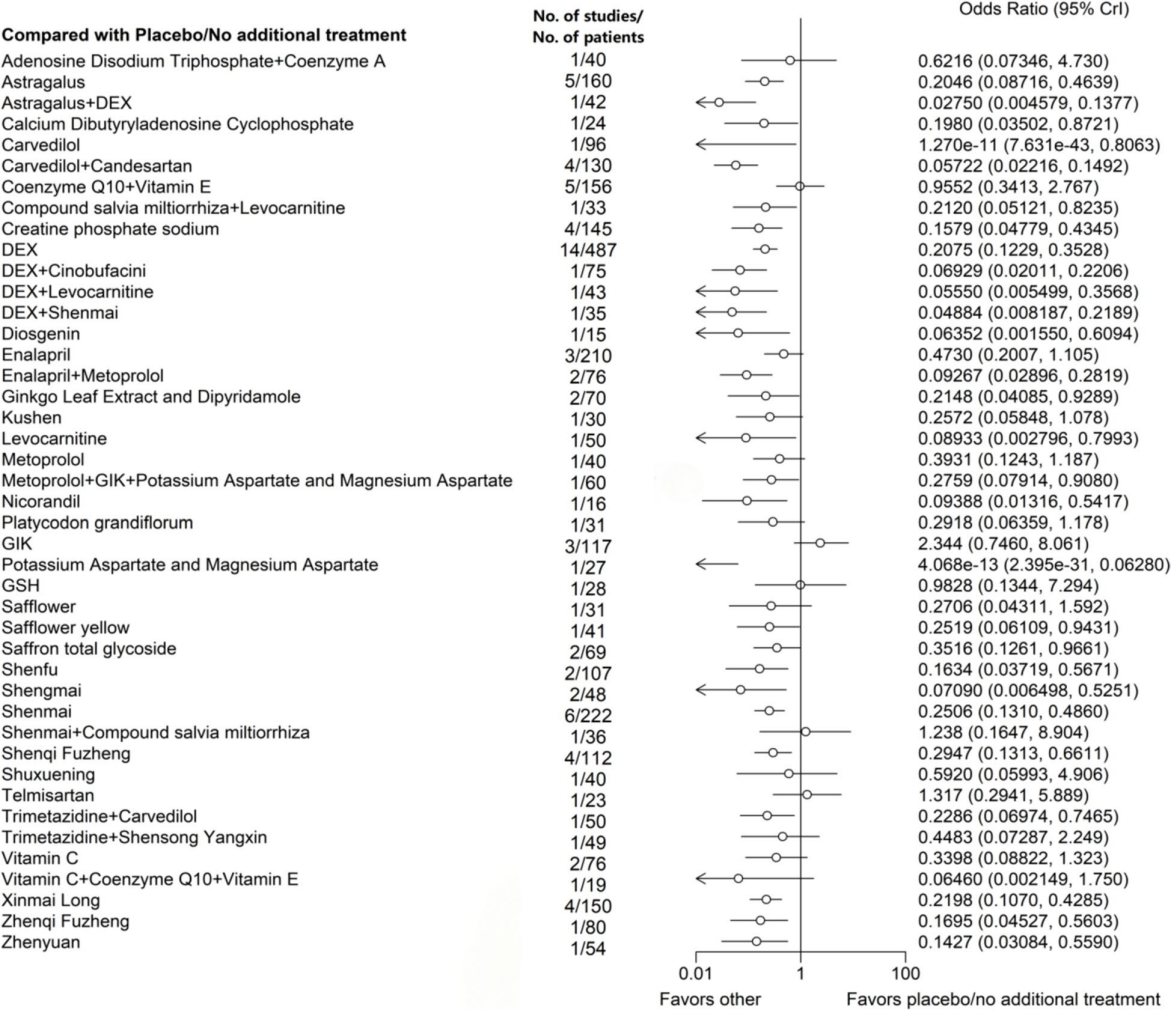
Network forest plot of electrocardiographic abnormalities. Note: DEX: Dexrazoxane; GSH: Glutathione; GIK: Glucose-insulin-potassium. Astragalus, Compound salvia miltiorrhiza, Cinobufacini, Diosgenin, Ginkgo Leaf Extract and Dipyridamole, Kushen, Platycodon grandiflorum, Safflower, Safflower yellow, Saffron total glycoside, Shenfu, Shengmai, Shenmai, Shenqi Fuzheng, Shuxuening, Shensong Yangxin, Xinmai Long, Zhenqi Fuzheng, and Zhenyuan are Chinese Patent Medicines, and most are injections (Diosgenin, Platycodon grandiflorum, Saffron total glycoside, Shensong Yangxin, Zhenqi Fuzheng, and Zhenyuan are solid oral dosage forms, including tablets, capsules, and granules)

A total of 28 treatments were found to be more effective than placebo/no treatment in preventing ECG abnormalities following anthracyclines use, except for the following: Adenosine Disodium Triphosphate + Coenzyme A (Odds Ratio [95% CrI], 0.6216 [0.0735, 4.730]), Coenzyme Q10 + Vitamin E (0.9552 [0.3413, 2.767]), Enalapril (0.4730 [0.2007, 1.105]), Kushen (0.2572 [0.05848, 1.078]), Metoprolol (0.3931 [0.1243, 1.187]), Platycodon grandiflorum (0.2918 [0.06359, 1.178]), GIK (2.344 [0.7460, 8.061]), Glutathione (GSH) (0.9828 [0.1344, 7.294]), Safflower (0.2706 [0.04311, 1.592]), Shenmai + Compound salvia miltiorrhiza (1.238 [0.1647, 8.904]), Shuxuening (0.5920 [0.05993, 4.906]), Telmisartan (1.317 [0.2941, 5.889]), Trimetazidine + Shensong Yangxin (0.4483 [0.07287, 2.249]), Vitamin C (0.3398 [0.08822, 1.323]), and Vitamin C + Coenzyme Q10 + Vitamin E (0.06460 [0.002149, 1.750]) (Fig. 4).

Based on the cumulative probability plot and SUCRA rankings, the highest-ranked treatment is Potassium aspartate and magnesium aspartate injection (SUCRA, 98.56%), followed by Carvedilol (93.38%), Astragalus + DEX (88.64%), DEX + Shenmai (82.62%), Carvedilol + Candesartan (82.14%), DEX + Levocarnitine (78.94%), DEX + Cinobufacini (78.52%), Shengmai (74.93%), and Diosgenin (74.27%) (Supplementary file 6).

#### Creatine Kinase-MB (CK-MB)

A total of 21 RCTs [40, 47, 48, 51, 52, 54, 61, 69, 76, 97, 101, 106, 108, 111, 115, 126, 127, 133, 136, 139, 142] reporting the serum levels of CK-MB before and after chemotherapy were included in the network meta-analysis. These studies evaluated 18 drug regimens (5 chemical drugs, 3 combined chemical and Chinese patent medicines, and 10 Chinese patent medicines) involving 1,933 patients who received anthracyclines (767 patients were randomly assigned to the placebo or no treatment group, while 1,166 patients were randomly assigned to the cardioprotective drug groups; see the Supplementary file 5 for the network relationship figure).

**Fig. 5:**
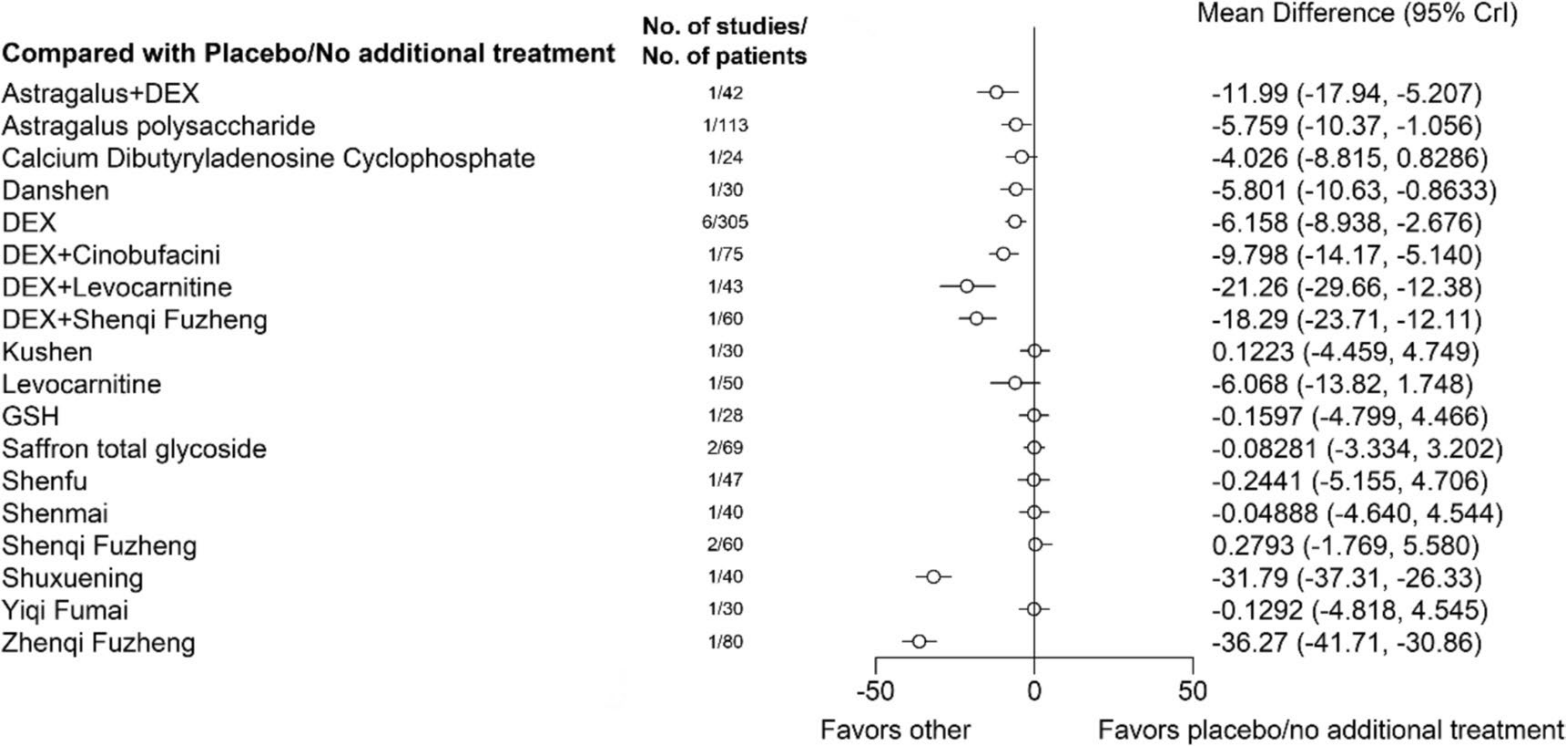
Network forest plot of CK-MB. Note: DEX: Dexrazoxane; GSH: Glutathione. Astragalus, Astragalus polysaccharide, Cinobufacini, Danshen, Kushen, Saffron total glycoside, Shenfu, Shenmai, Shenqi Fuzheng, Shuxuening, Yiqi Fumai, and Zhenqi Fuzheng are Chinese Patent Medicines, and most are injections (Saffron total glycoside, and Zhenqi Fuzheng are solid oral dosage forms, including tablets and granules)

For preventing an increase in serum CK-MB levels after chemotherapy, the following treatments had a beneficial effect compared to placebo/no treatment: Astragalus + DEX (Mean Difference [95% CrI], −11.99 [−17.94, −5.207]), Astragalus polysaccharide (−5.759 [−10.37, −1.056]), Danshen (−5.801 [−10.63, −0.8633]), DEX (−6.158 [−8.938, −2.676]), DEX + Cinobufacini (−9.798 [−14.17, −5.140]), DEX + Levocarnitine (−21.26 [−29.66, −12.38]), DEX + Shenqi Fuzheng (−18.29 [−23.71, −12.11]), Shuxuening (−31.79 [−37.31, −26.33]), and Zhenqi Fuzheng (−36.27 [−41.71, −30.86]) (Fig. 5).

Based on the cumulative probability plot and SUCRA rankings (Supplementary file 6), the top-ranked treatments are Zhenqi Fuzheng (SUCRA, 99.30%), Shuxuening (94.67%), DEX + Levocarnitine (87.05%), DEX + Shenqi Fuzheng (83.99%), Astragalus + DEX (75.02%), DEX + Cinobufacini (70.95%), followed by DEX (58.23%), Danshen (55.89%), Astragalus polysaccharide (55.87%), and Levocarnitine (55.47%).

#### Symptomatic cardiotoxicity

Forty-four RCTs [25, 26, 30–32, 40, 41, 44, 45, 50, 52–55, 57, 59, 60, 62, 64–66, 68, 77, 79, 80, 82, 84–87, 90, 97, 109, 111, 114, 115, 117, 121, 124, 130–133, 140] reporting symptomatic cardiac dysfunction in patients were included in the network meta-analysis. These studies evaluated 37 drug regimens (18 chemical drugs, 1 combined chemical and Chinese patent medicines, and 18 Chinese patent medicines) involving 3,862 patients who received anthracyclines (1,447 patients were randomly assigned to the placebo or no treatment group, while 2,415 patients were randomly assigned to the cardioprotective drug groups; see the Supplementary file 5 for the network relationship figure).

**Fig. 6:**
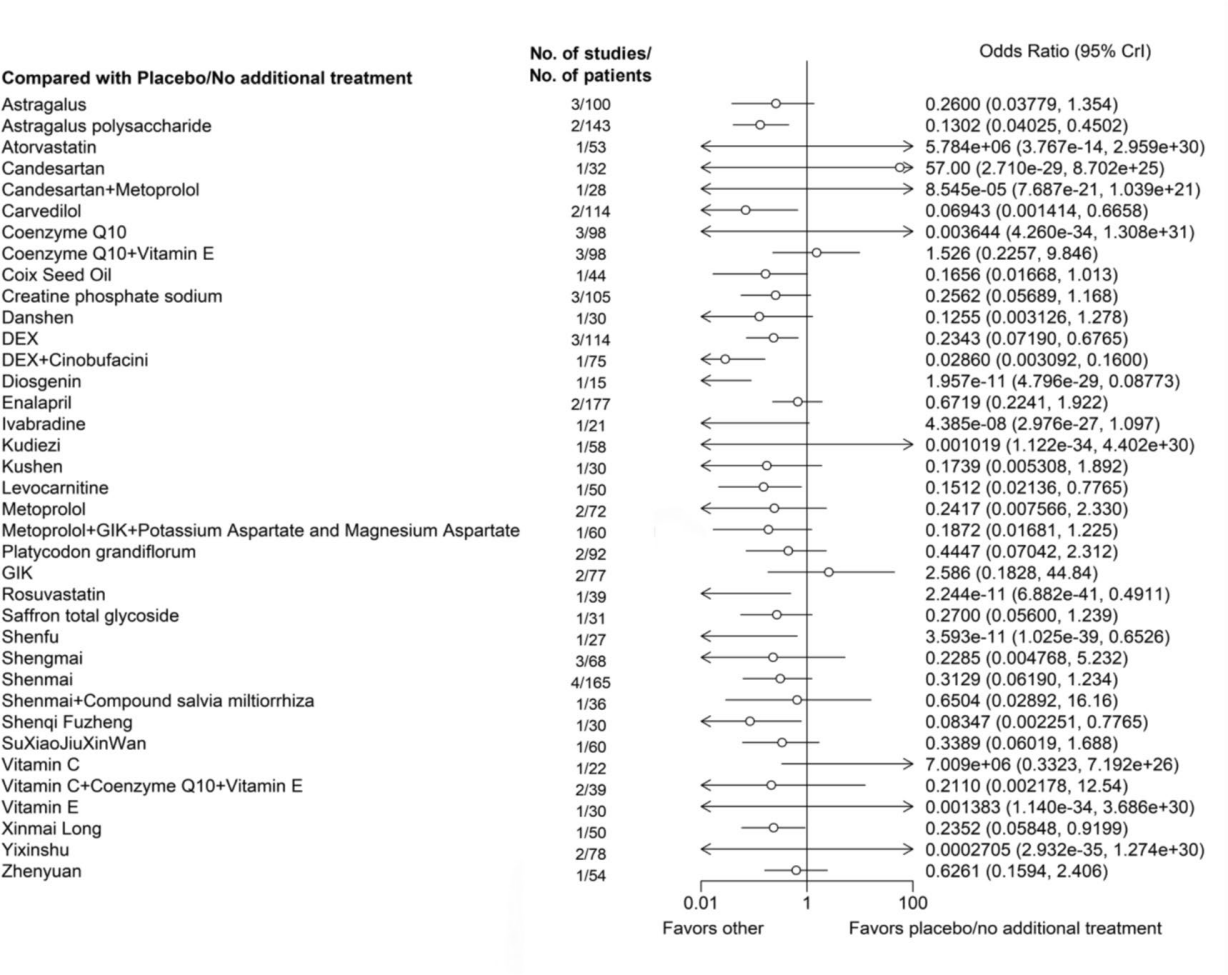
Network forest plot of symptomatic cardiotoxicity. Note: DEX: Dexrazoxane; GIK: Glucose-insulin-potassium. Astragalus, Astragalus polysaccharide, Coix Seed Oil, Compound salvia miltiorrhiza, Cinobufacini, Danshen, Diosgenin, Kudiezi, Kushen, Platycodon grandiflorum, Saffron total glycoside, Shenfu, Shengmai, Shenmai, Shenqi Fuzheng, SuXiaoJiuXinWan, Xinmai Long, Yiqi Fumai, and Zhenyuan are Chinese Patent Medicines, and most are injections (Diosgenin, Platycodon grandiflorum, Saffron total glycoside, SuXiaoJiuXinWan, and Zhenyuan are solid oral dosage forms, including tablets, capsules, and granules)

Regarding the prevention of symptomatic cardiac injury following anthracycline use, our results showed that the following treatments were more effective than placebo/no treatment: Astragalus polysaccharide (Odds Ratio [95% CrI], 0.1302 [0.04025, 0.4502]), Carvedilol (0.06943 [0.001414, 0.6658]), DEX (0.2343 [0.07190, 0.6765]), DEX + Cinobufacini (0.02860 [0.003092, 0.1600]), Diosgenin (0 [0, 0.08773]), Levocarnitine (0.1512 [0.02136, 0.7765]), Rosuvastatin (0 [0, 0.4911]), Shenfu (0 [0, 0.6526]), Shenqi Fuzheng (0.08347 [0.002251, 0.7765]), and Xinmai Long (0.2352 [0.05848, 0.9199]) (Fig. 6).

Based on the cumulative probability plot and SUCRA rankings, the highest-ranked treatment is Shenfu (SUCRA, 88.35%), followed by Rosuvastatin (87.84%), Diosgenin (87.04%), Ivabradine (84.86%), DEX + Cinobufacini (74.13%), Carvedilol (65.12%), Shenqi Fuzheng (61.90%), Candesartan + Metoprolol (59.11%), and Astragalus polysaccharide (58.68%) (Supplementary file 6).

#### Sensitivity analysis

In the fixed-effects model analysis, the point estimates for most treatments are stable. However, the narrowing of 95% CrIs indicates that some treatments such as Candesartan, Candesartan + Metoprolol, Enalapril, Lisinopril + Bisoprolol, Rosuvastatin, and Astragalus are significantly beneficial compared to placebo. Besides, no inconsistent evidence is detected in the sensitivity analysis for DEX and Carvedilo (Supplementary file 7).

### Treatment of anthracycline-induced cardiotoxicity

For patients who have already developed AIC, results from low-quality RCTs with small sample sizes have shown that Sacubitril/Valsartan Sodium, Levocarnitine, and Wenxin granules can achieve effectiveness rates of over 90%, significantly improving patients' symptoms and cardiac function. Additionally, Carvedilol has been found to increase LVEF in patients who experienced a decrease after anthracycline use (Table 2).

**Table 2:** RCTs Related to the Treatment of AIC

Study id	Age, y	Male sex n(%)	Anthracyclines	Treatments	No. patients	Duration of follow-up	Cardiac function		Response rate	Overall bias
							Prior treatment	Post treatment		
Chen RY, 2006 ^107^	25–68	-	Doxorubicin	Compound Danshen (Salvia miltiorrhiza) dripping pill + Vitamin C + Oryzanol + Vitamin B1; Vitamin C + Oryzanol + Vitamin B1	38; 26	15 days	-	-	89.47%; 69.23%	H
Li H., 2007 ^74^	32–72	51 (46.4%)	-	Sodium tanshinone IIA sulfonate + Astragalus injection + Vitamin E + Coenzyme Q_10_; Astragalus injection + Vitamin E + Coenzyme Q_10_	58; 52	14 days	-	-	Improvement of symptoms: 94.83%; 84.62% Electrocardiogram improvement: 87.93%; 80.77%	S
Li YQ, 2008a ^84^	48.35 ± 8.6	12 (33.3%)	Doxorubicin	Carvedilol; No additional treatment	18; 18	6 months	LVEDD 64.1 ± 9.8 mm; 63.89 ± 9.9 mm LVESD 51.3 ± 9.92 mm; 51.68 ± 9.68 mm LVEF 30.76 ± 8.1%; 31.02 ± 8.2%	LVEDD 57.26 ± 9.32 mm; 62.56 ± 9.89 mm LVESD 40.36 ± 9.65 mm; 49.02 ± 10.68 mm LVEF 44.78 ± 10.2%; 35.4 ± 10.1%	-	S
Wang LH, 2011 ^72^	13–71	33 (43.4%)	Daunorubicin/Doxorubicin/Mitoxantrone	Creatine phosphate sodium; Vitamin C	41; 35	7 days	CK-MB 33.2 ± 3.6 μmol/L; 34.1 ± 2.9 μmol/L LDH 172.5 ± 25.4 U/L; 159.6 ± 23.5 U/L HBDB 285.4 ± 49.3 U/L; 279.6 ± 51.3 U/L	CK-MB 21.3 ± 2.8 μmol/L; 32.5 ± 3.1 μmol/L LDH 112.4 ± 11.8 U/L; 139.2 ± 12.1 U/L HBDB 198.2 ± 45.2 U/L; 211.6 ± 39.6 U/L	-	S
Xu S, 2023 ^122^	50.4 ± 2.2	29 (43.9%)	-	Wenxin grannle; Placebo	33; 33	2 months	-	-	93.94%; 75.76%	S
Yan ZM, 2021 ^110^	52.9 ± 7.1	0 (0.0%)	Doxorubicin	Sacubitril/Valsartan sodium; No additional treatment	35; 35	3 months	LVEDD 42.10 ± 2.28 mm; 42.19 ± 2.14 mm LVEF 61.59 ± 3.47%; 61.48 ± 3.77%	LVEDD 40.94 ± 1.84 mm; 41.03 ± 1.41 mm LVEF 61.98 ± 3.01%; 62.67 ± 3.21%	94.29%; 74.29%	S
Zhang LQ, 2015 ^135^	4.7 ± 1.7	33 (55.0%)	-	Levocarnitine; Vitamin C + Coenzyme Q10 + Vitamin E	30; 30	/	CK 491 ± 75 U/L; 493 ± 79 U/L	CK 245 ± 57 U/L; 286 ± 53 U/L	93.33%; 80.00%	S
Zhou AM, 2011 ^143^	31–74	39 (43.3%)	-	Amiodarone; Wenxin grannle	45; 45	2 months	-	-	82.22%; 95.56%	S

## Discussion

Anthracyclines are a crucial component in the treatment of cancer, and their safety has been a topic of ongoing concern. Their severe cardiotoxicity limits the use of anthracycline-based chemotherapy regimens, especially in patients with high cardiovascular risk. Our study included 128 RCTs related to the prevention and treatment of AIC, involving 10,431 patients who received anthracyclines and 78 drug regimens (41 chemical drugs, 10 combined chemical and Chinese patent medicines, and 27 Chinese patent medicines).

A Bayesian network meta-analysis was conducted using a random-effects model, incorporating seventy-two RCTs that reported LVEF. The results indicated that, compared to no treatment, patients treated with Calcium Dibutyryladenosine Cyclophosphate, Carvedilol, Carvedilol + Candesartan, Compound Salvia Miltiorrhiza + Levocarnitine, DEX, DEX + Cinobufacini, DEX + Shenqi Fuzheng, Nicorandil, Qiliqiangxin, and Xinmai Long showed less variation in LVEF following anthracycline use. The SUCRA ranking results indicated that the most effective treatment option for preserving LVEF was Nicorandil (SUCRA 91.76%). However, it should be noted that the findings for Calcium Dibutyryladenosine Cyclophosphate, Compound Salvia Miltiorrhiza + Levocarnitine, DEX + Cinobufacini, DEX + Shenqi Fuzheng, Nicorandil, and Qiliqiangxin are derived from a single small-sample RCT.

Our study found that although they all belong to β-blockers, Carvedilol (Mean Difference [95% CrI], 4.024 [0.5372, 7.656]) can prevent the decline of LVEF after anthracycline use, while Metoprolol (0.2373 [−5.150, 5.671]), Nebivolol (−0.003988 [−7.998, 7.941]), and Lisinopril + Bisoprolol (5.199 [−3.580, 14.03]) do not appear to have protective effects against AIC. Our finding aligns with He, D. et al. [148], indicating Carvedilol's superiority over Metoprolol, Bisoprolol, and Nebivolol in preventing LVEF decline after anthracycline use, with Metoprolol showing no significant difference from placebo. Recent RCTs and meta-analyses have consistently supported the positive effects of Carvedilol on AIC [13, 149].

The exact mechanism of AIC remains unclear. Oxidative stress, which promotes the formation of large amounts of reactive oxygen species (ROS), and the inhibition of topoisomerase IIβ in cardiomyocytes leading to mitochondrial defects, are relatively well-accepted mechanisms [150, 151]. Carvedilol may mitigate AIC by reducing ROS release, inhibiting cell apoptosis, and preventing lipid peroxidation in cardiomyocytes [152, 153].

Nicorandil showed the highest efficacy for LVEF improvement in SUCRA rankings (SUCRA 91.76%), but its single small-sample RCT basis necessitates larger-scale validation. DEX's protective effects on AIC are supported by numerous studies [8], and our study further confirms its positive effects. Additionally, our study found that the combination of DEX with some Chinese patent medicines may be more effective than DEX alone (DEX + Shenqi Fuzheng [SUCRA 90.05%] > DEX + Cinobufacini [87.54%] > DEX [62.06%]). Exploring the mechanisms of the potential cardioprotective effects of these Chinese patent medicines and validating their efficacy through further multicenter, large-sample clinical trials could be promising directions for future research.

Our study also found that, compared to placebo, Candesartan and Telmisartan (ARBs), Enalapril (ACEIs), and Eplerenone (MRAs) did not prevent the decline in LVEF. Moreover, Enalapril and Telmisartan failed to significantly mitigate electrocardiographic abnormalities, while Candesartan and Enalapril did not significantly prevent symptomatic cardiotoxicity, as compared to placebo/no treatment. Histological studies have shown that AT_1a_-mediated Ang II signaling contributes to doxorubicin-induced cardiac injury [154], exacerbating fibrosis and dysfunction in mice exposed to doxorubicin [155]. Existing RCTs have not found significant differences between ACEIs/ARBs versus placebo/no treatment in preventing AIC, but this does not exclude the possibility of insufficient sample size and statistical power affecting the results.

Statins have shown promising results in preclinical models. They can reduce the incidence of oxidative stress, cardiomyocyte death, and cardiac contractile dysfunction through inhibiting 3-hydroxy-3-methylglutaryl-coenzyme A reductase and activating endothelial nitric oxide synthase [156–158]. However, the results of RCTs and meta-analyses on whether statins can improve patients’ LVEF and prevent AIC are controversial [159–162]. Our study shows that there is not enough evidence to prove that statins (Atorvastatin and Rosuvastatin) have a positive effect on preventing the decline in LVEF, and more large-scale RCTs based on hard endpoints are needed to determine the efficacy of statins in preventing AIC.

In addition, Sildenafil, Ivabradine, Levocarnitine, N-acetylcysteine, and Glutathione have not shown beneficial effects on preventing the decline in LVEF. Coenzyme Q10, Vitamin E, and Vitamin C have failed to demonstrate efficacy in preventing the decline in LVEF, electrocardiographic abnormalities, and symptomatic cardiotoxicity.

For the treatment of patients who have already developed AIC, there is currently insufficient evidence to support the beneficial effects of any drugs. Preliminary findings from small-sample, low-quality RCTs hint at potential improvements in cardiac function with Sacubitril/Valsartan Sodium, Levocarnitine, Wenxin Granules, and Carvedilol. However, these observations are hampered by the limited number and quality of studies, necessitating confirmation through larger-scale, rigorously designed RCTs with low risk of bias.

This extensive network meta-analysis, unprecedentedly comprising 128 RCTs involving 10,431 patients and evaluating 78 distinct drug regimens, is the largest network meta-analysis conducted on the prevention and treatment of AIC. This is the greatest advantage and innovation of our study. However, there are also several limitations: 1) Regarding heterogeneity, four intervention pairs demonstrated high heterogeneity (I^2^ > 75%) in the LVEF, and one pair in the CK-MB (Supplementary file 4). Potential reasons include bias in study implementation and outcome measurement/assessment, notably the failure of many studies to report blinding of participants, researchers, and outcome assessors, as well as the instruments and methods used for measuring LVEF and CK-MB. Furthermore, due to the limited number of studies and incomplete data (specifically, the omission of anthracycline types and doses in certain studies), it was impractical to further evaluate the influence of sample size, anthracycline types and doses, follow-up duration, and tumor types on heterogeneity using quantitative methods such as subgroup analysis. Considering potential unquantifiable confounding factors influencing the study outcomes, we pre-determined to use the random-effects model for pooling effect sizes, owing to its superior ability to accommodate uncertainty and offer broader confidence intervals than the fixed-effects model. 2) Apart from DEX, almost all other treatments have been evaluated in ≤ 5 RCTs, and more than half in a single RCT. Consequently, the planned meta-regression analyses, stratified by follow-up duration, sample size, and anthracycline type, could not be executed as intended; 3) Given the small number of studies (1–3) for most treatments, methods to assess publication bias, such as funnel plots, Begg's test, and Egger's test, were not employed. This limitation underscores the potential for publication bias in our findings; 4) The overall quality of the included studies was moderate, with 84.4% assessed as having "some concerns". Furthermore, the majority of treatments were evaluated in a limited number of low- and moderate-quality studies with small sample sizes, necessitating confirmation of our findings through large-scale, higher-quality studies; 5) Besides, by focusing solely on Chinese and English literature, this study may have inadvertently excluded relevant studies published in other languages, introducing a potential source of language bias. Future endeavors should strive for a more comprehensive, multilingual approach to mitigate this limitation.

## Conclusions

Apart from DEX, Carvedilol, a β-blocker, also appears to show significant potential in preventing AIC. Furthermore, our results indicate that there is insufficient evidence to support the beneficial effects of statins (Atorvastatin and Rosuvastatin), Sildenafil, Ivabradine, Levocarnitine, N-acetylcysteine, Glutathione, Coenzyme Q10, Vitamin E, and Vitamin C in preventing LVEF decline and exerting a positive effect on the prevention of AIC. However, due to the limited number of studies and small sample sizes for most treatments, our findings await further confirmation by high-quality, large-scale RCTs.

## Supplementary Information

Supplementary Material 1.

Supplementary Material 2.

Supplementary Material 3.

Supplementary Material 4.

Supplementary Material 5.

Supplementary Material 6.

Supplementary Material 7.

## Data Availability

The original contributions presented in the study are included in the article/supplementary material, further inquiries can be directed to the corresponding authors.

## Abbreviations

RCTs: Randomized Controlled Trials
AIC: Anthracycline-induced Cardiotoxicity
LVEF: Left Ventricular Ejection Fraction
SUCRA: Surface Under Cumulative Ranking Curve
DEX: Dexrazoxane
GIK: Glucose-Insulin-Potassium
GSH: Glutathione
ECG: Electrocardiogram
CK-MB: Creatine Kinase-MB

## References

[1] Venkatesh P, Kasi A. Anthracyclines. In: StatPearls. Treasure Island (FL) ineligible companies. Disclosure: Anup Kasi declares no relevant financial relationships with ineligible companies.: StatPearls Publishing Copyright © 2024, StatPearls Publishing LLC.; 2024.

[2] Khan AA, Ashraf A, Singh R, et al. Incidence, time of occurrence and response to heart failure therapy in patients with anthracycline cardiotoxicity. Intern Med J. 2017;47(1):104–9. 27800661 10.1111/imj.13305

[3] Herrmann J. Adverse cardiac effects of cancer therapies: cardiotoxicity and arrhythmia. Nat Rev Cardiol. 2020;17(8):474–502. 32231332 10.1038/s41569-020-0348-1 PMC8782611

[4] Lotrionte M, Biondi-Zoccai G, Abbate A, et al. Review and meta-analysis of incidence and clinical predictors of anthracycline cardiotoxicity. Am J Cardiol. 2013;112(12):1980–4. 24075281 10.1016/j.amjcard.2013.08.026

[5] Cardinale D, Colombo A, Bacchiani G, et al. Early detection of anthracycline cardiotoxicity and improvement with heart failure therapy. Circulation. 2015;131(22):1981–8. 25948538 10.1161/CIRCULATIONAHA.114.013777

[6] Macedo AVS, Hajjar LA, Lyon AR, et al. Efficacy of dexrazoxane in preventing anthracycline cardiotoxicity in breast cancer. JACC CardioOncology. 2019;1(1):68–79. 34396164 10.1016/j.jaccao.2019.08.003 PMC8352186

[7] Tebbi CK, London WB, Friedman D, et al. Dexrazoxane-associated risk for acute myeloid leukemia/myelodysplastic syndrome and other secondary malignancies in pediatric Hodgkin’s disease. J Clin Oncol. 2007;25(5):493–500. 17290056 10.1200/JCO.2005.02.3879

[8] de Baat EC, Mulder RL, Armenian S, et al. Dexrazoxane for preventing or reducing cardiotoxicity in adults and children with cancer receiving anthracyclines. Cochrane Datab Syst Rev. 2022;9(9):Cd014638. 10.1002/14651858.CD014638.pub2 PMC9512638 36162822

[9] Shaikh F, Dupuis LL, Alexander S, Gupta A, Mertens L, Nathan PC. Cardioprotection and second malignant neoplasms associated with dexrazoxane in children receiving anthracycline chemotherapy: a systematic review and meta-analysis. J National Cancer Instit. 2016;108(4):djv357. 10.1093/jnci/djv357 26598513

[10] Mohamed AL, El-Abd AA, Mohamed HG, Noufal AM, Hennawy BS. Therapy in prevention of anthracycline-induced cardiotoxicity: a three dimentional echocardiography study. Curr Problems Cardiol. 2024;Part C. 49(1):(no pagination)(102130). 10.1016/j.cpcardiol.2023.102130 37858847

[11] Bhasin V, Vakilpour A, Scherrer-Crosbie M. Statins for the primary prevention of anthracycline cardiotoxicity: a comprehensive review. Curr Oncol Rep. 2024;26(10):1197–204. 39002055 10.1007/s11912-024-01579-6 PMC11480194

[12] Ma Y, Bai F, Qin F, et al. Beta-blockers for the primary prevention of anthracycline-induced cardiotoxicity: a meta-analysis of randomized controlled trials. BMC Pharmacol Toxicol. 2019;20(1):18. 31023386 10.1186/s40360-019-0298-6 PMC6485127

[13] Zhan T, Daniyal M, Li J, Mao Y. Preventive use of carvedilol for anthracycline-induced cardiotoxicity: a systematic review and meta-analysis of randomized controlled trials. Herz. 2020;45(Suppl 1):1–14. 30656389 10.1007/s00059-018-4779-y

[14] Li J, Lee A, Tariq A, et al. Comparing renin-angiotensin-aldosterone blockade regimens for long-term chemotherapy-related cardiac dysfunction: a network meta-analysis. Cardiovasc Drugs Ther. 2023;39(1):171–86. 37314568 10.1007/s10557-023-07457-w

[15] Gao Y, Wang R, Jiang J, Hu Y, Li H, Wang Y. ACEI/ARB and beta-blocker therapies for preventing cardiotoxicity of antineoplastic agents in breast cancer: a systematic review and meta-analysis. Heart Fail Rev. 2023;28(6):1405–15. 37414918 10.1007/s10741-023-10328-z PMC10575808

[16] Li C, Zheng Y, Niu D, et al. Effects of Traditional Chinese Medicine Injections for Anthracyclines-induced Cardiotoxicity: An Overview of Systematic Reviews and Meta-Analyses. Integr Cancer Ther. 2023;22:15347354231164752. 37057304 10.1177/15347354231164753 PMC10108409

[17] Liberati A, Altman DG, Tetzlaff J, et al. The PRISMA statement for reporting systematic reviews and meta-analyses of studies that evaluate healthcare interventions: explanation and elaboration. BMJ (Clinical research ed). 2009;339:b2700. 19622552 10.1136/bmj.b2700 PMC2714672

[18] Hutton B, Salanti G, Caldwell DM, et al. The PRISMA extension statement for reporting of systematic reviews incorporating network meta-analyses of health care interventions: checklist and explanations. Ann Intern Med. 2015;162(11):777–84. 26030634 10.7326/M14-2385

[19] Miller Ab Fau - Hoogstraten B, Hoogstraten B Fau - Staquet M, Staquet M Fau - Winkler A, Winkler A. Reporting results of cancer treatment. Cancer. 1981;47(0008-543X (Print)):207-214. 10.1002/1097-0142(19810101)47:1<207::aid-cncr2820470134>3.0.co;2-6 7459811

[20] Sterne JAC, Savović J, Page MJ, et al. RoB 2: a revised tool for assessing risk of bias in randomised trials. BMJ (Clinical research ed). 2019;366:l4898. 31462531 10.1136/bmj.l4898

[21] Abuosa AM, Elshaikh AH, Qureshi K, et al. Prophylactic use of carvedilol to prevent ventricular dysfunction in patients with cancer treated with doxorubicin. Eur J Heart Fail. 2018;20(200):2018–2005. 10.1016/j.ihj.2018.06.011 PMC6310701 30595329

[22] Acar Z, Kale A, Turgut M, et al. Efficiency of atorvastatin in the protection of anthracycline-induced cardiomyopathy. J Am Coll Cardiol. 2011;58(9):988–9. 21851890 10.1016/j.jacc.2011.05.025

[23] Armenian SH, Hudson MM, Lindenfeld L, et al. Effect of carvedilol versus placebo on cardiac function in anthracycline-exposed survivors of childhood cancer (PREVENT-HF): a randomised, controlled, phase 2b trial. Lancet Oncol. 2024;25(2):235–245p. 38215764 10.1016/S1470-2045(23)00637-X PMC10872217

[24] Attar A, Heydari M, Abtahi F, et al. Sildenafil for primary prevention of anthracycline-induced cardiac toxicity: a phase I/II randomized clinical trial, SILDAT-TAHA6 trial. Cardiol Res Pract. 2022;2022(no pagination):5681510. 35387238 10.1155/2022/5681510 PMC8977337

[25] Avila MS, Ayub-Ferreira SM, de Barros Wanderley MR, et al. Carvedilol for prevention of chemotherapy-related cardiotoxicity: the CECCY trial. J Am Coll Cardiol. 2018;71(20):2281–90. 29540327 10.1016/j.jacc.2018.02.049

[26] Ciburiene E, Aidietiene S, Scerbickaite G, et al. Ivabradine for the prevention of anthracycline-induced cardiotoxicity in female patients with primarily breast cancer: a prospective, randomized, open-label clinical trial. Medicina. 2023;59(12):2140. 38138243 10.3390/medicina59122140 PMC10745010

[27] Cochera F, Dinca D, Bordejevic DA, et al. Nebivolol effect on doxorubicin-induced cardiotoxicity in breast cancer. Cancer Manag Res. 2018;10:2071–81. 30038521 10.2147/CMAR.S166481 PMC6053261

[28] Davis MK, Villa D, Tsang TSM, Starovoytov A, Gelmon K, Virani SA. Effect of eplerenone on diastolic function in women receiving anthracycline-based chemotherapy for breast cancer. JACC Cardiooncol. 2019;1(2):295–298p. 34396193 10.1016/j.jaccao.2019.10.001 PMC8352029

[29] Dessi M, Piras A, Madeddu C, et al. Long-term protective effects of the angiotensin receptor blocker telmisartan on epirubicin-induced inflammation, oxidative stress and myocardial dysfunction. Eur J Cancer. 2011;47:2011–2009. 10.3892/etm.2011.305 PMC3440822 22977612

[30] Georgakopoulos P, Kyriakidis M, Perpinia A, et al. The role of metoprolol and enalapril in the prevention of doxorubicin-induced cardiotoxicity in lymphoma patients. Anticancer Res. 2019;39(10):5703–7. 31570470 10.21873/anticanres.13769

[31] Gulati G, Heck SL, Ree AH, et al. Prevention of cardiac dysfunction during adjuvant breast cancer therapy (PRADA): A 2 x 2 factorial, randomized, placebo-controlled, double-blind clinical trial of candesartan and metoprolol. Eur Heart J. 2016;37(21):1671–80. 26903532 10.1093/eurheartj/ehw022 PMC4887703

[32] Hao W, Shi Y, Qin Y, et al. Platycodon grandiflorum protects against anthracycline-induced cardiotoxicity in early breast cancer patients. Integ Cancer Ther. 2020;19:1534735420945017. 10.1177/1534735420945017 PMC7491211 32729334

[33] Henriksen PA, Hall P, MacPherson IR, et al. Multicenter, prospective, randomized controlled trial of high-sensitivity cardiac troponin i-guided combination angiotensin receptor blockade and beta-blocker therapy to prevent anthracycline cardiotoxicity: the cardiac CARE trial. Circulation. 2023;148(21):1680–1690p. 37746692 10.1161/CIRCULATIONAHA.123.064274 PMC10655910

[34] Hundley WG, D’Agostino R Jr, Crotts T, et al. Statins and left ventricular ejection fraction following doxorubicin treatment. NEJM Evid. 2022;1(9):EVIDoa2200097. 10.1056/evidoa2200097 PMC9997095 36908314

[35] Jhorawat R, Kumari S, Varma SC, et al. Preventive role of carvedilol in adriamycin-induced cardiomyopathy. Indian J Med Res. 2016;144(5):725–729p. 28361826 10.4103/ijmr.IJMR_1323_14 PMC5393084

[36] Jo SH, Kim LS, Kim SA, et al. Evaluation of short-term use of N-acetylcysteine as a strategy for prevention of anthracycline-induced cardiomyopathy: EPOCH trial - a prospective randomized study. Korean Circul J. 2013;43(3):174–81. 10.4070/kcj.2013.43.3.174 PMC3629243 23613694

[37] Kalay N, Basar E, Ozdogru I, et al. Protective effects of carvedilol against anthracycline-induced cardiomyopathy. J Am Coll Cardiol. 2006;48(11):2258–62. 17161256 10.1016/j.jacc.2006.07.052

[38] Lee M, Chung WB, Lee JE, et al. Candesartan and carvedilol for primary prevention of subclinical cardiotoxicity in breast cancer patients without a cardiovascular risk treated with doxorubicin. Cancer Med. 2021;10(12):3964–73. 33998163 10.1002/cam4.3956 PMC8209607

[39] Li C, Zhou X, Song X. Protective effects of traditional Chinese medicine injections against anthracycline-induced cardiac toxicity. Anti-Tumor Pharm. 2013;3(5):385–388p.

[40] Li X, Guo X, Li J, Yuan L, Wang H. Preventing effect of astragalus polysaccharide on cardiotoxicity induced by chemotherapy of epirubicin: a pilot study. Medicine. 2022;101(32):e30000p. 35960075 10.1097/MD.0000000000030000 PMC9371539

[41] Nabati M, Janbabai G, Esmailian J, Yazdani J. Effect of rosuvastatin in preventing chemotherapy-induced cardiotoxicity in women with breast cancer: a randomized, single-blind, placebo-controlled trial. J Cardiovasc Pharmacol Ther. 2019;24(3):233–41. 30599756 10.1177/1074248418821721

[42] Osataphan N, Phrommintikul A, Leemasawat K, et al. Effects of metformin and donepezil on the prevention of doxorubicin-induced cardiotoxicity in breast cancer: a randomized controlled trial. Sci Rep. 2023;13(1):12759. 37550350 10.1038/s41598-023-40061-4 PMC10406870

[43] Rahimi K, Amoozgar H, Zareifar S, et al. Cardioprotective effects of deferoxamine in acute and subacute cardiotoxicities of doxorubicin: a randomized clinical trial. Egypt Heart J. 2023;75(1):21. 36961611 10.1186/s43044-023-00347-4 PMC10039151

[44] Slowik AJ, Jagielski P, Potocki P, et al. Anthracycline-induced cardiotoxicity prevention with angiotensin-converting enzyme inhibitor ramipril in women with low-risk breast cancer: Results of a prospective randomized study. Kardiol Pol. 2020;78(2):131–7. 31995035 10.33963/KP.15163

[45] Thavendiranathan P, Houbois C, Marwick TH, et al. Statins to prevent early cardiac dysfunction in cancer patients at increased cardiotoxicity risk receiving anthracyclines. Eur Heart J Cardiovasc Pharmacother. 2023;9(6):515–25. 37120736 10.1093/ehjcvp/pvad031 PMC10509566

[46] Wihandono A, Azhar Y, Abdurahman M, Hidayat S. The role of lisinopril and bisoprolol to prevent anthracycline induced cardiotoxicity in locally advanced breast cancer patients. Asian Pac J Cancer Prev. 2021;22(9):2847–2853p. 34582653 10.31557/APJCP.2021.22.9.2847 PMC8850900

[47] Jiang ZH, Zhang GJ, Xu LL, Cao ZY. Effects of dexrazoxane combined with huangqi injection in preventing and treating adriamycin-induced cardiotoxicity. Int J Lab Med. 2018;39(11):412–22.

[48] Chen BB. Clinical study on the prevention and treatment of anthracycline-induced cardiotoxicity with yiqi fumai injection (Freeze-Dried) [Master's Thesis]. 2016.

[49] Zhong L, Chen FC. The Protective effect of dexrazoxane against epirubicin-induced cardiotoxicity in breast cancer patients. Med Equip. 2017;30(22):406–12.

[50] Chen HL. Observation on the prevention of anthracycline-induced cardiotoxicity with Shenmai injection as the main treatment. Zhejiang J Trad Chinese Med. 2009;44(12):103053.

[51] Chen JT. Clinical study on preventing acute cardiotoxicity induced by anthracyclines with the method of supplementing qi to strengthen the body [Master's Thesis]. 2015.

[52] Zhuo XJ, Shi LX, Chen LZ. Effect of Danshen injection on preventing cardiotoxicity induced by pirarubicin or epirubicin chemotherapy. Chinese Hospital Pharmaceut J. 2018;18(4):1047700.

[53] Ning YL, Yao SL, Han HT, Chen X. Preliminary Study on the Effect of Wei-Ao-Xin Tablet Against Adriamycin-Induced Cardiotoxicity. Chinese Journal of Cardiovascular Rehabilitation Medicine. 2005;14(4).

[54] Chen ZY. Clinical study on the prevention of anthracycline-induced cardiotoxicity in breast cancer patients with crocin tablets [Master's Thesis]. 2022.

[55] Ren HH, Cui XJ. Clinical observation on the prevention of anthracycline-induced cardiotoxicity with Shenmai injection. Chinese Foreign Med Res. 2012;31(15):103053.

[56] Liu WJ, Li GF, Zhai Q. Effect of high-dose Vitamin C in preventing adriamycin-induced cardiotoxicity. Chinese J Clin Oncol. 1997;2(2):23.

[57] Yang JQ, Dong ZQ. Clinical observation on the prevention of anthracycline-related cardiotoxicity with Huangqi injection. Chinese Commun Doc (Medical Specialty). 2010;12(31):23.

[58] Xu X, Liu W, Duan XB. Study on the preventive effect of xinmailong injection on anthracycline-induced cardiotoxicity. Chinese Med Inform Herald. 2016;8(4):17–26.

[59] Wen CH, Xu XH, Zhou ZT, Lu MQ, Feng XQ. Clinical observation on the prevention of anthracycline chemotherapy-related cardiac adverse reactions with coix seed oil injection. Oncology. 2009;29(10):593.

[60] Sun YS, Gao DR. Efficacy of coenzyme Q10 in treating anthracycline-induced cardiotoxicity. Modern Oncol. 2012;20(10):4418–24.

[61] Wang SY, Wang XC, Chen SY, Wang WL, Lv JY, Gao WB. A multicenter prospective clinical study on the prevention of anthracycline-induced cardiotoxicity in elderly patients with Shenqi Fuzheng injection. J Inner Mongolia Univ Nationalities (Natural Sciences Edition). 2020;35(4):5800575.

[62] Yang SL, Wang MS, Lang LX, et al. Clinical Study on the Prevention of Daunorubicin-Induced Cardiotoxicity in Acute Leukemia Patients with Xinmailong Injection. Chinese Journal of Integrated Traditional Chinese and Western Medicine on Cardio-Cerebrovascular Diseases. 2019;17(20).

[63] Shao Y, Han F. Clinical observation on the prevention of epirubicin-induced cardiotoxicity in breast cancer patients with Qili Qiangxin capsules. Cardiovasc Dis Prevent Control Knowledge (Academic Edition). 2023;13(4):e30000.

[64] Hao W. Clinical efficacy observation of platycodon grandiflorum in preventing anthracycline-induced cardiotoxicity in breast cancer patients: A randomized controlled double-blind trial [Master’s Thesis]. Shanghai Univ Trad Chinese Med. 2016;19:1534735420945017.

[65] Hao W. Preventive effect of Shenmai injection on adriamycin-induced cardiotoxicity. Clin J Trad Chinese Med. 2007;19(1):23.

[66] He MW. Clinical study on the prevention of acute adriamycin-induced cardiotoxicity with Shengmai injection [Master's Thesis]. 2006.

[67] Zhao F, Wang J, Zhang R, Xu WX, He CY. Prevention and treatment of anthracycline-induced cardiotoxicity by dexrazoxane. China Pharmaceut J. 2015;24(7):2862.

[68] Zhou GH, Cao D, Guo HY, Hu XC. Clinical observation on the prevention of doxorubicin-related cardiotoxicity with Huangqi injection. Chin J Cancer. 2005;15(3):23.

[69] Lu ZH, Zong Q, Chen SL, Bu XG, Hu YQ. Clinical study on the treatment of adriamycin-induced cardiotoxicity with shuxuening. J Clin Exper Med. 2014;13(22):249–61.

[70] Li S, Hua Q. Clinical study on the treatment of ventricular arrhythmia induced by adriamycin cardiotoxicity with trimetazidine. Chin Commun Doc (Medical Specialty). 2007;9(20):e23084.

[71] Huang LQ, Wu JY, Li SL. Preventive effect of nicorandil on pirarubicin-induced cardiotoxicity during chemotherapy. J Qilu Med College. 2015;30(06):644–6.

[72] Wang LH, Guo PX, He Y, Huang ZY. Efficacy of sodium phosphocreatine injection in treating anthracycline-induced cardiotoxicity. Chin J Gerontol. 2011;31(18):103053.

[73] Li G, Liu LN, Liu L, Zhang NX, Ji YH. Clinical study on the prevention of arrhythmia induced by anthracycline chemotherapy in breast cancer patients with enalapril and metoprolol. Capital Food Med. 2021;28(2):5703–7.

[74] Li H, Jia WQ. Treatment of chemotherapy-induced cardiotoxicity with tanshinone IIA sulfonate. J Pharmaceut Forum. 2007;28(1):1267525.

[75] Yang J, Jiang WZ. Analysis of the preventive effect of trimetazidine combined with low-dose carvedilol on cardiotoxicity after anthracycline chemotherapy in breast cancer patients. J Clin Exper Med. 2018;17(1):936–40.

[76] Zhao L, Ge YN, Jing QM. Study on the prevention of cardiotoxicity induced by anthracyclines in postoperative breast cancer patients with Dexrazoxane. Oncol Pharm. 2019;9(5):68–79.

[77] Wang D, Liu YF, Zhang JF, Li B. Clinical observation on the prevention of acute cardiotoxicity induced by anthracyclines in children with acute leukemia treated with sodium phosphocreatine. Mater Child Health Care China. 2013;28(14):598708.

[78] Gu B, Li C. Observation on the treatment of adriamycin-induced cardiotoxicity with Shenqi Fuzheng injection. Basic Clin Oncol. 2011;24(5):1592.

[79] Wang CJ, Chen QQ, Deng L, Lu QH, Liao ZJ, Li JJ. Clinical observation on the prevention of anthracycline chemotherapy-induced cardiotoxicity with Shenmai injection. China J Trad Med Sci Technol. 2003;10(2):100936.

[80] Li Q, Yang LD. Clinical study on the protective effect of sodium phosphocreatine on adriamycin-induced cardiotoxicity. Modern Oncology. 2013;21(3):608–10.

[81] Li SL, Hao YL. Prevention and Mechanism of Ginkgo Dipyridamole Injection on Anthracycline-Induced Cardiotoxicity. Paper presented at: The Second Qi Huang Forum of China Association of Chinese Medicine — Blood Disease Prevention and Treatment Sub-Forum 2014; Beijing, China.

[82] Hu XB, Song SF, Li XQ. Clinical observation on the prevention of adriamycin-related cardiotoxicity with Yueanxin. Occupation Health. 2004;20(4):e23084.

[83] He JC, Li XQ. Exploring the preventive effect of xinmailong injection on anthracycline-induced cardiotoxicity. Northern Pharm. 2016;13(8):444.

[84] Li YQ. Observation on the therapeutic effect of carvedilol in the treatment of heart failure caused by adriamycin-induced cardiomyopathy. J Mudanjiang Med College. 2008;29(3):725–9.

[85] Li YQ. Clinical observation on the treatment of adriamycin-induced myocardial damage in breast cancer patients with Huangqi injection. Heilongjiang Med J. 2008;31(5):23.

[86] Kong JX, Su XC, Yan BC, Liang BS. Clinical observation on the prevention of anthracycline-induced cardiotoxicity with astragalus polysaccharide injection. Xin Zhong Yi (New Chinese Medicine). 2013;45(7):444.

[87] Liang JK, Teng SW. Clinical Observation on the Prevention of Adriamycin-Induced Cardiotoxicity with Quick-Acting Heart-Rescuing Pills. Journal of Traditional Chinese Medicine; 2000; Hangzhou.

[88] Cui YZ, Zhang SJ, Han YG, Liang XW. Clinical observation on the prevention of anthracycline-induced cardiotoxicity with Shenqi Fuzheng injection. Hebei Med. 2011;33(11):5800575.

[89] Cai XZ, Huang LN, Chen JP, Lin CP. Observation on the preventive effect of dexrazoxane on epirubicin-induced cardiotoxicity. Pharmaceut J Today. 2013;23(1):e5228.

[90] Lin WB. Observation on the effect of dexrazoxane in preventing epirubicin-induced cardiotoxicity. Modern Diagnos Treat. 2017;28(6):e5228.

[91] Liu JZ. Clinical effect of sodium phosphocreatine in preventing acute cardiotoxicity induced by anthracyclines in children with acute leukemia. Chin J Clin Oncol Rehab. 2015;22(6):12418–25.

[92] Wang JH, Zhou H, He NX, Liu XK. Clinical comparative study on the prevention of adriamycin-induced cardiotoxicity with vitamin C (Report of 30 Cases). Guizhou Med J. 1996;20(4):23.

[93] Liu XM, Huang YH. Prospective study on the prevention of pirarubicin-induced cardiotoxicity with Dexrazoxane. Jiangxi Med J. 2016;51(03):246–9.

[94] Liu YJ. Analysis of toxicity and prevention of anthracycline chemotherapy in the treatment of breast cancer. China Med Guide. 2020;18(20):15–23.

[95] Liu Y. Clinical observation on the prevention of adriamycin-related cardiotoxicity with safflower yellow pigment injection. Chin Commun Doc. 2008;24(23):e23084.

[96] Chen R, Qiu SD, Chen F, Pang Y, Liu JH. Echocardiographic evaluation of the preventive effect of dexrazoxane on daunorubicin-induced cardiotoxicity. J Pract Med. 2012;28(24):257–65.

[97] Li XP, Zheng LZ, Zhang L, Gu JC, Ma F. Clinical observation on the prevention of epirubicin-related cardiotoxicity with Kushen injection. J Oncol. 2010;16(5):395–7.

[98] Li X, Liu W, Niu B. Preventive value of low-dose carvedilol combined with candesartan on anthracycline-induced cardiotoxicity in breast cancer patients. Practical Oncol J. 2015;30(2):256–9.

[99] Li XF, Wang HX, Pei Y. Clinical observation on the prevention of epirubicin-induced cardiotoxicity with shensong yangxin capsule combined with trimetazidine. Chinese Remed Clin. 2014;14(10):1447–9.

[100] Yang J, He JL, Zou YH, An XR, Zhang GR, Qu HW. Preventive effect of candesartan combined with carvedilol on anthracycline-induced cardiotoxicity. Tianjin Pharmaceut J. 2016;28(4):31–3.

[101] Miao YD, Quan WX. Clinical study on the prevention of subacute cardiotoxicity after doxorubicin chemotherapy with dibutyryl cyclic adenosine monophosphate calcium. Clin Focus. 2017;32(12):1053–6.

[102] Wang CY, Song CY, Shen FM. Treatment of cardiotoxicity with shenmai injection in 31 cases. Shaanxi J Trad Chinese Med. 2013;34(2):150–1.

[103] Shen N. Clinical observation on the prevention of anthracycline-induced cardiotoxicity with Shenfu injection. Chin J Emerg Med. 2010;19(7):1132–3.

[104] Kong JX, Su XC, Dai SL, Deng YM, Cheng L, Song J. Clinical observation on the prevention of anthracycline-induced cardiotoxicity with astragalus polysaccharide injection. China Med Herald. 2017;14(20):136–9.

[105] Chen CM, Tang FM, Jiang Z, Chen GP, Mo SF, Su XR. Clinical study on the prevention of arrhythmia induced by anthracycline cardiotoxicity with enalapril and metoprolol. Chin Med Innov. 2020;17(11):31–6.

[106] Su Y. Effect of dexrazoxane combined with L-carnitine in preventing cardiotoxicity induced by epirubicin chemotherapy in liver cancer patients. J Clin Med Res. 2020;37(8):1139–42

[107] Chen RY, Zhao YX, Zhao XS, Sun P. Observation on the therapeutic effect of compound danshen dripping pills in treating cardiotoxicity induced by doxorubicin chemotherapy in malignant tumors. Chin J Appropriate Technol Diagnosis Ther. 2006;24(1):455–6.

[108] Sun CY, Yang SL, Hou W, Wang MS, Li YH, Wang YS. Clinical observation on the prevention of daunorubicin-induced myocardial toxicity in acute myeloid leukemia patients with Shenmai injection. Shanghai J Trad Chin Med. 2012;46(8):47–9.

[109] Dong HP, Tong LM. Clinical observation on the prevention of epirubicin-related cardiotoxicity with Yixinshu Capsule. Inner Mongolia J Trad Chin Med. 2011;30(1):40–1.

[110] Yan ZM, Wang L. Clinical Observation on the therapeutic effect of sacubitril/valsartan sodium in treating anthracycline-induced myocardial damage. Shaanxi Med J. 2021;50(6):1–8.

[111] Wang QY. Clinical observation on the prevention of anthracycline-related cardiotoxicity with Qizhengfu injection in 30 cases. China Med Guide. 2012;10(15):1242596.

[112] Wang X, Chen JY, Xiong D, He XD, Yang B. Preventive effect of dexrazoxane on anthracycline-induced cardiotoxicity. Clin Med Lit Elect J. 2016;3(25):4989–90.

[113] Wang X. Study on the prevention of cardiotoxicity during chemotherapy in acute leukemia patients with dexrazoxane combined with Shenmai injection. Chin J Rehab Med. 2015;27(12):412–22.

[114] Wang Y. Antagonistic effect of zhenyuan capsule on adriamycin-induced cardiotoxicity during ovarian chemotherapy. Chinese J Misdiagnosis. 2005;5(4):705–6.

[115] Wang YM, Xu L, Wang GF, Jiang CY, Lu HZ. Cardioprotective effect of L-carnitine in arterial infusion chemotherapy for elderly patients with liver malignancies. Liver. 2011;16(4):319–20.

[116] Chen ZS, Chen X, Ouyang XN, Cheng HH, Wei W. Clinical study on the prevention of adriamycin-induced cardiotoxicity with potassium aspartate and magnesium aspartate. J Clin Oncol. 1998;3(4):129–36.

[117] Dong JH, Zhang Y, Su XG, Wu L. Clinical observation on the prevention of anthracycline chemotherapy-induced cardiotoxicity with shenmai injection combined with Xiangdan injection. Modern J Integ Trad Chin Western Med. 2007;16(2):100936.

[118] Qi C, Zhou ZZ, Wang Y, Zhang X, Xiang DH. Clinical study on the prevention of adriamycin-induced cardiotoxicity with Shexiang Baoxin Pill. Modern Oncol. 2017;25(20):759.

[119] Liu L, Liu ZZ, Liu YY, et al. Role of low-dose carvedilol combined with candesartan in preventing anthracycline-induced cardiotoxicity during adjuvant chemotherapy for breast cancer. Chin J Oncol. 2013;35(12):1095–110. 24506965

[120] Fan LD, Xu FJ. Evaluation of the preventive effect of dexrazoxane on epirubicin-induced cardiotoxicity in breast cancer patients. Beijing Med J. 2016;38(10):406–12.

[121] Zhang YK, He MW, Zhang H, He HY, Xie J, Xu JP. Observation on the therapeutic effect of shengmai injection in preventing acute adriamycin-induced cardiotoxicity in 40 cases. Chin J Emerg Med. 2007;16(9):759.

[122] Xu S. Analysis of clinical treatment effect on arrhythmia induced by anthracycline antitumor drugs. Healthy Women. 2023;38(19–20):22.

[123] Liu W, Duan XB, Xu X. Study on the preventive effect of xinmailong injection on anthracycline-induced cardiotoxicity. Chin Gen Pract. 2014;17(29):5800575.

[124] Xu ZL, Zhang W, Sun L, Qiu H, Zhang C. Clinical observation on the treatment of advanced breast cancer with shenfu injection combined with Epirubicin and Paclitaxel. Clin Med Pract (Second Half Monthly). 2008;1(8):648–9.

[125] Xue XP. Prevention and treatment of acute anthracycline-induced cardiotoxicity in elderly patients. Chin J Misdiagnosis. 2011;11(13):14–27.

[126] Zhang HB, Geng H, Jiao XY, Gao SL, Yan XT. Clinical study on the prevention of adriamycin-induced cardiotoxicity with Zhenqi Fuzheng granules. Shanxi Med J (First Half). 2012;41(5):759.

[127] Yang MD, Zhang PC, Zhang X, Zhao JJ, Zhang Y, Yan DX. Observation on the preventive effect of dexrazoxane on epirubicin-induced cardiotoxicity in breast cancer patients undergoing epirubicin chemotherapy. Shandong Med J. 2022;62(15):406–12.

[128] Yu MJ, Yang RH. Clinical study on the prevention of anthracycline-induced cardiotoxicity in breast cancer patients with icardozolin injection. Chin J Clin Pharmacol. 2022;38(7):14–27.

[129] Jiang YQ, Song GP, Zheng LX, et al. Clinical study on the prevention of adriamycin-related cardiotoxicity with safflower injection. Modern J Integ Trad Chin Western Med. 2010;19(25):3146–7.

[130] Yang XL. Clinical observation on the prevention of doxorubicin-related cardiotoxicity with Shengmai injection. China Med Guide. 2008;6(15):e2308.

[131] Wu SN, Chen WL, Yang Z. Effect of enalapril in preventing cardiotoxicity during anthracycline and trastuzumab combination therapy in breast cancer patients. Chin J Cardiol. 2023;28(6):256–9.

[132] Zhai ZW, Yuan Y. Therapeutic effect of metoprolol plus potassium magnesium polarized liquid in preventing adriamycin-induced cardiotoxicity. J Xinxiang Med Univ. 2007;24(2):152–3.

[133] Zhang GW. Effect of dexrazoxane alone or combined with cinobufagin injection on adriamycin-induced cardiotoxicity in acute cancer patients. Pract Oncol J. 2016;31(7):412–22.

[134] Zhang JP, Tian F. Clinical observation on the prevention of adriamycin-induced cardiotoxicity with Huangqi injection. Paper presented at: 2009 International Conference on Oncology in Chinese Medicine 2009; Tianjin, China.

[135] Zhang LQ, Qu XF, Zhu XJ. Clinical observation on the prevention of anthracycline antitumor drug-induced cardiotoxicity with L-carnitine in 30 cases. Global Trad Chin Med. 2015;8(S1):252.

[136] Gong YL, Zhang QY. Observation on the prevention of epirubicin-induced cardiotoxicity with reduced glutathione. Chin J Rehab Theory Pract. 2008;14(8):e30000.

[137] Zhang XJ. Preventive effect of sodium phosphocreatine on adriamycin-induced cardiotoxicity in leukemia children undergoing chemotherapy. Frontier of Medicine. 2019;9(25):63–4.

[138] Wu BR, Chen LJ, Zhang XF. Clinical observation on the prevention of epirubicin-induced cardiotoxicity with dexrazoxane. J Taishan Med Univ. 2015;36(11):406–12.

[139] Zhang YC. Clinical observation on the prevention of anthracycline chemotherapy-induced cardiotoxicity with crocin in breast cancer patients [Master's Thesis]. 2022.

[140] Zhang YC, Guan N, Li L, Lu YY. Clinical observation on the prevention of daunorubicin-induced cardiotoxicity with dexrazoxane in 43 cases. China Health Care Nutr. 2020;30(9):34–5.

[141] Zhao BF. Preventive and therapeutic effect of dexrazoxane on pirarubicin-induced cardiotoxicity. Capital Food Med. 2017;24(6):26.

[142] Chen JG, Wang ZD, Shi L, Liang C, Zhao JX. Clinical observation on the prevention of anthracycline-induced cardiotoxicity with Shenfu injection. Chin J Emerg Med. 2009;18(12):103053.

[143] Zhou AM. Clinical observation on the therapeutic effect of wenxin granule in treating arrhythmia induced by anthracycline antitumor drugs. Oncol Pharm. 2011;1(06):535–7.

[144] Zhou C, Xie F, Zhang N, Fei Q, Wang Y. Clinical evaluation of compound danshen combined with L-carnitine injection in protecting myocardial injury induced by adriamycin chemotherapy in breast cancer patients. Chin J Metallurg Industry Med. 2017;34(4):476–7.

[145] Li YD, Wang W, Zhou L. Observation on the preventive effect of ginkgo dipyridamole injection on anthracycline chemotherapy-induced cardiotoxicity. Chin J Pract Intl Med. 2006;26(S1):1067–77.

[146] Zhou Y. Application effect of L-carnitine in the treatment of anthracycline-induced cardiotoxicity in breast cancer patients. Clin Rational Drug Use J. 2021;14(6):190–206.

[147] Zou Y. Preventive effect of xinmailong injection on anthracycline-induced cardiotoxicity. Chin J Integ Trad Chin Western Med Cardio Cerebrovasc Dis. 2017;15(20):100936.

[148] He D, Hu J, Li Y, Zeng X. Preventive use of beta-blockers for anthracycline-induced cardiotoxicity: a network meta-analysis. Front Cardiovasc Med. 2022;9:968534. 36035937 10.3389/fcvm.2022.968534 PMC9403514

[149] Kheiri B, Abdalla A, Osman M, et al. Meta-analysis of carvedilol for the prevention of anthracycline-induced cardiotoxicity. Am J Cardiol. 2018;122(11):1959–64. 30292333 10.1016/j.amjcard.2018.08.039

[150] Kuang Z, Ge Y, Cao L, et al. Precision treatment of anthracycline-induced cardiotoxicity: an updated review. Curr Treat Options Oncol. 2024;25(8):1038–54. 39066853 10.1007/s11864-024-01238-9 PMC11329674

[151] Narezkina A, Narayan HK, Zemljic-Harpf AE. Molecular mechanisms of anthracycline cardiovascular toxicity. Clin Sci (London, England : 1979). 2021;135(10):1311–32. 10.1042/CS20200301 PMC10866014 34047339

[152] Cheng J, Kamiya K, Kodama I. Carvedilol: molecular and cellular basis for its multifaceted therapeutic potential. Cardiovasc Drug Rev. 2001;19(2):152–71. 11484068 10.1111/j.1527-3466.2001.tb00061.x

[153] Spallarossa P, Garibaldi S, Altieri P, et al. Carvedilol prevents doxorubicin-induced free radical release and apoptosis in cardiomyocytes in vitro. J Mol Cell Cardiol. 2004;37(4):837–46. 15380674 10.1016/j.yjmcc.2004.05.024

[154] Toko H, Oka T, Zou Y, et al. Angiotensin II type 1a receptor mediates doxorubicin-induced cardiomyopathy. Hypertens Res. 2002;25(4):597–603. 12358147 10.1291/hypres.25.597

[155] Agostinucci K, Grant MKO, Seelig D, et al. Divergent cardiac effects of angiotensin II and isoproterenol following juvenile exposure to doxorubicin. Front Cardiovasc Med. 2022;9:742193. 35402534 10.3389/fcvm.2022.742193 PMC8990895

[156] Sharma H, Pathan RA, Kumar V, Javed S, Bhandari U. Anti-apoptotic potential of rosuvastatin pretreatment in murine model of cardiomyopathy. Int J Cardiol. 2011;150(2):193–200. 20452068 10.1016/j.ijcard.2010.04.008

[157] Ramanjaneyulu SV, Trivedi PP, Kushwaha S, Vikram A, Jena GB. Protective role of atorvastatin against doxorubicin-induced cardiotoxicity and testicular toxicity in mice. J Physiol Biochem. 2013;69(3):513–25. 23385671 10.1007/s13105-013-0240-0

[158] Dadson K, Thavendiranathan P, Hauck L, et al. Statins protect against early stages of doxorubicin-induced cardiotoxicity through the regulation of akt signaling and SERCA2. CJC open. 2022;4(12):1043–52. 36562012 10.1016/j.cjco.2022.08.006 PMC9764135

[159] Titus A, Cheema HA, Shafiee A, et al. Statins for attenuating cardiotoxicity in patients receiving anthracyclines: a systematic review and meta-analysis. Curr Probl Cardiol. 2023;48(10):101885. 37336312 10.1016/j.cpcardiol.2023.101885

[160] D’Amario D, Laborante R, Bianchini E, et al. Statins as preventive therapy for anthracycline cardiotoxicity: a meta-analysis of randomized controlled trials. Int J Cardiol. 2023;391:131219. 37527752 10.1016/j.ijcard.2023.131219

[161] Agarwal S, Guha A, Krishan S, et al. Statins for primary prevention of anthracycline chemotherapy-related cardiac dysfunction: a systematic review and meta-analysis of randomized controlled trials. Am J Cardiol. 2023;206:63–6. 37683579 10.1016/j.amjcard.2023.08.123 PMC11345853

[162] Felix N, Nogueira PC, Silva IM, et al. Cardio-protective effects of statins in patients undergoing anthracycline-based chemotherapy: An updated meta-analysis of randomized controlled trials. Eur J Intern Med. 2024;126:43–8. 38643042 10.1016/j.ejim.2024.04.007

