# Supplementary material for: Prevention and treatment of anthracycline-induced cardiotoxicity: a systematic review and network meta-analysis of randomized controlled trials
Source: Cardiooncology. 2025 Jul 10;11:66. doi: 10.1186/s40959-025-00360-3 (PMC12243438; doi:10.1186/s40959-025-00360-3)
Supplement: Supplementary file 1 — Supplementary Material 1. [file 40959_2025_360_MOESM1_ESM.docx]

Prevention and Treatment of Anthracycline-Induced Cardiotoxicity: A Systematic Review and Network Meta-analysis of Randomized Controlled Trials

Current Oncology Reports

Siyu Li, MD^a¶^, Wenrui Li, MD ^a¶^, Mengfei Cheng, MD ^a^, Xiaoxiao Wang, PhD ^a^, [Wanyi Chen](http://www.frontiersin.org/Community/WhosWhoActivity.aspx?sname=WanyiChen&UID=2784852), PhD ^a^*

Affiliations

1. Department of Pharmacy, Chongqing University Cancer Hospital, Chongqing, China.

¶These authors contributed equally to this work.

* Corresponding author

E-mail: [chenwanyi@cqu.edu.cn](mailto:chenwanyi@cqu.edu.cn) (WCh)

Present address: No. 181, Hanyu Road, Shapingba District, Chongqing, China.

## Search Strategy

1 exp anthracycline antibiotic agent/ or exp anthracycline/ or exp anthracycline derivative/

2 exp daunorubicin/

3 exp doxorubicin/

4 exp epirubicin/

5 exp pirarubicin/

6 exp aclarubicin/

7 exp idarubicin/

8 exp valrubicin/

9 exp mitoxantrone/

10 exp cardiotoxicity/

11 exp heart arrhythmia/

12 exp heart muscle ischemia/

13 exp cardiomyopathy/

14 exp heart failure/

15 exp major adverse cardiac event/

16 (cardiotoxicity or arrhythmia or "myocardial ischemia" or "heart muscle ischemia" or cardiomyopathy or "cardiac failure" or "heart failure" or "adverse cardiac event" or "adverse cardiac events" or "adverse cardiac reaction" or "adverse cardiac effect" or "adverse cardiac effects").kw,ti.

17 (anthracycline or daunorubicin or doxorubicin or epirubicin or pirarubicin or aclarubicin or Idarubicin or valrubicin or mitoxantrone).kw,ti.

18 (treatment or prevention or management).kw,ti.

19 1 or 2 or 3 or 4 or 5 or 6 or 7 or 8 or 9

20 10 or 11 or 12 or 13 or 14 or 15

21 17 or 19

22 16 or 20

23 18 and 21 and 22
