# Supplementary material for: Prevention and treatment of anthracycline-induced cardiotoxicity: a systematic review and network meta-analysis of randomized controlled trials
Source: Cardiooncology. 2025 Jul 10;11:66. doi: 10.1186/s40959-025-00360-3 (PMC12243438; doi:10.1186/s40959-025-00360-3)
Supplement: Supplementary file 2 — Supplementary Material 2. [file 40959_2025_360_MOESM2_ESM.docx]

Prevention and Treatment of Anthracycline-Induced Cardiotoxicity: A Systematic Review and Network Meta-analysis of Randomized Controlled Trials

Current Oncology Reports

Siyu Li, MD^a¶^, Wenrui Li, MD ^a¶^, Mengfei Cheng, MD ^a^, Xiaoxiao Wang, PhD ^a^, [Wanyi Chen](http://www.frontiersin.org/Community/WhosWhoActivity.aspx?sname=WanyiChen&UID=2784852), PhD ^a^*

Affiliations

1. Department of Pharmacy, Chongqing University Cancer Hospital, Chongqing, China.

¶These authors contributed equally to this work.

* Corresponding author

E-mail: [chenwanyi@cqu.edu.cn](mailto:chenwanyi@cqu.edu.cn) (WCh)

Present address: No. 181, Hanyu Road, Shapingba District, Chongqing, China.

Table 1 The characteristics of studies

| Study id | Country | Age, y | Male sex, n(%) | Anthracyclines | Treatments | Number of patients | Duration of follow-up | Outcomes | Overall bias |
| --- | --- | --- | --- | --- | --- | --- | --- | --- | --- |
| Abuosa, A. M., 2018 | Saudi Arabia | 42.5±15.3 | 92 (59.7%) | Doxorubicin | Carvedilol; Placebo | 116; 38 | 6 months | LVEDD; LVEF; LVESD | L |
| Acar, Z., 2011 | Turkey | 53.2±16.0 | 17 (42.5%) | Adriamycin/  Idarubicin | Atorvastatin; No additional treatment | 20; 20 | 6 months | LVEDD; LVEF; LVESD | S |
| Armenian, S. H., 2024 | United States and Canada | 27.5±10.1 | 79 (52.3%) | / | Carvedilol; Placebo | 75; 76 | 2 years | BNP; E/A; FS; LVEDD; LVEDV; LVEF; LVESD; LVESV; LVM; LVESWS; NT-proBNP; Troponin; Z score | L |
| Attar, A., 2022 | Iran | 42.2±14.9 | 15 (28.8%) | / | Sildenafil; Placebo | 24; 28 | 6 months | GLS; LVEDD; LVEDV; LVEF; LVESD; LVESV; Number of cardiotoxicities; Troponin | L |
| Avila, M. S., 2018 | St. Paulo, Brazil | 51.9±9.6 | 0 (0.0%) | Doxorubicin | Carvedilol; Placebo | 96; 96 | 6 months | BNP; ECG abnormality; LVEDD; LVEF; LVESD; Number of cardiotoxicities; Troponin | L |
| Cai XZ, 2013 | China | 38–73 | 15 (25.0%) | Epirubicin | Dexrazoxane; No additional treatment | 30; 30 | 4.2 months | ECG abnormality; LVEF; Troponin | S |
| Chen BB, 2016 | China | 51.7±4.3 | 11 (18.3%) | Doxorubicin/  Epirubicin | Yiqi Fumai Lyophilized injection; No additional treatment | 30; 30 | 2.8 months | CK-MB; LVEF; NT-proBNP; Troponin | S |
| Chen CM, 2020 | China | / | 0 (0.0%) | Epirubicin | Enalapril; Metoprolol; Enalapril + Metoprolol; No additional treatment | 40; 40; 40; 40 | 4.2 months | CK; CK-MB; ECG abnormality | S |
| Chen HL, 2009 | China | 30–72 | 24 (27.9%) | Doxorubicin | Shenmai injection; Coenzyme Q10 + Vitamin E | 45; 41 | 1 months | AST; CK; ECG abnormality; LDH; LVEF; Number of cardiotoxicities | S |
| Chen JG, 2009 | China | 31–73 | 37 (40.2%) | / | Shenfu injection; No additional treatment | 47; 45 | 10 days | CK; CK-MB; ECG abnormality; HBDH；LDH; Number of cardiotoxicities; Response rate | S |
| Chen JT, 2015 | China | 44.3±9.6 | 36 (60.0%) | / | Shenqi Fuzheng injection; No additional treatment | 30; 30 | 21 days | BNP; CK; CK-MB; ECG abnormality; LDH; LVEF; Troponin | S |
| Chen R, 2012 | China | 25–68 | / | Doxorubicin | Compound Danshen (Salvia miltiorrhiza) dripping pill + Vitamin C + Oryzanol + Vitamin B1; Vitamin C + Oryzanol + Vitamin B1 | 38; 26 | 15 days | ECG abnormality; Response rate | H |
| Chen RY, 2006 | China | 41.0±11.9 | 19 (47.5%) | Daunorubicin | Dexrazoxane; No additional treatment | 20; 22 | 2.1 months | E/A; LVEF; Tei index | S |
| Chen ZS, 1998 | China | 23–73 | 34 (64.2%) | Doxorubicin | Potassium aspartate and magnesium aspartate injection; No additional treatment | 27; 26 | / | ECG abnormality | S |
| Chen ZY, 2022 | China | 51.0±9.0 | 0 (0.0%) | Epirubicin | Saffron total glycoside tablet; No additional treatment | 31; 29 | 2.8 months | BNP; CK; CK-MB; ECG abnormality; LVEF; Number of cardiotoxicities; Troponin | S |
| Ciburiene, E., 2023 | Vilnius, Lithuania | 47.9±10.1 | 0 (0.0%) | Doxorubicin/  Epirubicin | Ivabradine; No additional treatment | 21; 27 | 6 months | GLS; LVEF; NT-proBNP; Number of cardiotoxicities; Troponin | S |
| Cochera, F., 2018 | Romania | 52.5±12.1 | 0 (0.0%) | Doxorubicin | Nebivolol; No additional treatment | 30; 30 | 3.7±0.4 months | E/A; FS; IVRT; LVEDD; LVEF; LVESD; MAPSE;Number of cardiotoxicities | S |
| Cui YZ, 2011 | China | 33–62 | 0 (0.0%) | / | Shenqi Fuzheng injection; No additional treatment | 22; 20 | 3 months | ECG abnormality; LVEF | S |
| Davis, M. K., 2019 | Canada | 51.7±9.2 | 0 (0.0%) | Doxorubicin | Eplerenone; Placebo | 22; 19 | 6 months | LVEF; Number of cardiotoxicities | L |
| Dessi, M., 2011 | Italy | 52.9±9.5 | 12 (24.5%) | Epirubicin | Telmisartan; Placebo | 23; 21 | 12 months | DecT; E/A; ECG abnormality; Em; Em/Am; LVEF; Sm | S |
| Dong HP, 2011 | China | 45.2±46.2 | 29 (50.0%) | Epirubicin | Yixinshu; Coenzyme Q10 | 30; 28 | 2.8 months | E/A; ECG abnormality; FS; LVEF; LVEDD; LVESD; Number of cardiotoxicities | S |
| Dong JH, 2007 | China | 28–66 | 41 (56.2%) | Doxorubicin | Shenmai injection + Compound salvia miltiorrhiza injection; Polarized solution (Glucose-insulin-potassium, GIK) | 36; 37 | 2.8 months | CK; ECG abnormality; LDH; Number of cardiotoxicities; Troponin | S |
| Fan LD, 2016 | China | 20–65 | 0 (0.0%) | Epirubicin | Dexrazoxane; No additional treatment | 106; 94 | 2.8 months | BNP; LVEF; Troponin | S |
| Georgakopoulos, P., 2010 | Athens, Greece | 49.2±17.9 | 65 (52.0%) | Doxorubicin | Metoprolol; Enalapril; No additional treatment | 42; 43; 40 | 31 months | E/A; E/Ea; FS; LVEDD; LVEF; LVESD; Number of cardiotoxicities | H |
| Gong YL, 2008 | China | 51.0±5.9 | 0 (0.0%) | Epirubicin | Reduced glutathione (GSH); No additional treatment | 28; 28 | 4.2 months | CK; CK-MB; ECG abnormality; Em/Am; FS; LDH; LVEF | S |
| Gu B, 2011 | China | 30–6l | 0 (0.0%) | Doxorubicin | Shenqi Fuzheng injection; No additional treatment | 30; 30 | 21 days | ECG abnormality; Troponin | S |
| Gulati, G., 2016 | Norway | 50.8±9.6 | 0 (0.0%) | Epirubicin | Candesartan + Metoprolol; Candesartan; Metoprolol; Placebo | 28; 32; 30; 30 | 2.5 months - 15.25 months | LVEF; Number of cardiotoxicities | L |
| Hao W, 2007 | China | 28–70 | 29 (48.3%) | Doxorubicin | Shenmai injection; No additional treatment | 30; 30 | 2.1 months | Number of cardiotoxicities | S |
| Hao W, 2016 | China | 50.5±10.7 | 0 (0.0%) | Epirubicin | Platycodon grandiflorum ; Placebo | 31; 31 | 2.1 months | ECG abnormality; LVEF; Number of cardiotoxicities; Troponin | L |
| Hao, W., 2020 | Shanghai, China | 49.5±10.7 | 0 (0.0%) | Doxorubicin | Platycodon Grandiflorum; Placebo | 61; 64 | 2 years | LVEF; Number of cardiotoxicities | L |
| He JC, 2016 | China | 51.4±3.5 | 45 (56.3%) | / | Xinmai Long injection; No additional treatment | 40; 40 | 4.2 months | E/A; ECG abnormality; Troponin | S |
| He MW, 2006 | China | 50.9±14.3 | 28 (75.7%) | Doxorubicin | Shengmai injection; Vitamin C + Coenzyme Q10 + Vitamin E | 18; 19 | 8 days | CK-MB; ECG abnormality; Number of cardiotoxicities; Troponin | S |
| Henriksen, P. A., 2023 | Britain | 54.0±13.5 | 45 (78.9%) | Epirubicin | Carvedilol + Candesartan; No additional treatment | 27; 27 | 6 months | ECG abnormality; GCS; GLS; LAA;LVEDV; LVEF; ; Number of cardiotoxicities; Troponin | H |
| Hu XB, 2004 | China | 40.1±6.5 | 27 (27.6%) | Doxorubicin | Kudiezi injection; Coenzyme Q10 | 58; 40 | / | CK; ECG abnormality; LDH; Number of cardiotoxicities | S |
| Huang LQ, 2015 | China | 32–70 | 22 (68.8%) | Pirarubicin | Nicorandil; No additional treatment | 16; 16 | 4.2 months | ECG abnormality; LVEF; Troponin | S |
| Hundley, W. G., 2022 | United States | 49±12.0 | 23 (8.2%) | Doxorubicin | Atorvastatin; Placebo | 100; 105 | 2 years | LVEDV; LVEF; LVESV; LVM; LVMI; Troponin; SV | S |
| Jhorawat, R., 2016 | Chandigarh, India | 41.3±17.3 | 41 (75.9%) | Doxorubicin | Carvedilol; No additional treatment | 27; 27 | 6 months | A; E; E/A; IVCT; IVRT; LVEDD; LVEF; LVESD | S |
| Jiang YQ, 2010 | China | 34–68 | 19 (32.2%) | Doxorubicin | Safflower injection; Coenzyme Q10 + Vitamin E | 31; 28 | / | A/E; ECG abnormality; FS; LVEDD; LVEF; LVESD | S |
| Jiang ZH, 2018 | China | 44.5±9.3 | 41 (51.3%) | Doxorubicin | Astragalus injection + Dexrazoxane; Dexrazoxane | 42; 38 | 2.8 months | CK-MB; ECG abnormality; LVEF; Troponin | S |
| Jo, S. H., 2013 | Korea | 49.8±9.2 | 5 (4.9%) | Doxorubicin/  Epirubicin | N-acetylcysteine; Placebo | 47; 52 | 12 months | A; CK; CK-MB; E; E/A; LVEDD; LVEF; LVESD; Troponin | S |
| Kalay, N., 2006 | Turkey | 47.9±12.1 | 7 (14.0%) | Doxorubicin/  Epirubicin | Carvedilol; Placebo | 25; 25 | 6 months | A; E; E/A; IVCT; IVRT; LVEDD; LVEF; LVESD | S |
| Kong JX, 2013 | China | 20–63 | 27 (45.0%) | Epirubicin/  Pirarubicin | Astragalus polysaccharide injection; Creatine phosphate sodium | 30; 30 | 2.8 months | BNP; LVEF; Number of cardiotoxicities; Troponin | S |
| Kong JX, 2017 | China | 46.5±11.4 | 30 (37.5%) | Epirubicin/  Pirarubicin | Astragalus polysaccharide injection; Creatine phosphate sodium | 40; 40 | 2.8 months | BNP; LVEF | S |
| Lee, M., 2021 | Korea | 47.2±8.2 | 0 (0.0%) | Doxorubicin | Candesartan; Carvedilol | 82; 70 | 12 months | E/A; LVEDD; LVEF; LVESD; Number of cardiotoxicities | S |
| Li G, 2021 | China | 52.7±4.1 | 0 (0.0%) | / | Enalapril + Metoprolol; Enalapril | 36; 36 | 2.8 months | CK; CK-MB; ECG abnormality | S |
| Li H, 2007 | China | 32–72 | 51 (46.4%) | / | Sodium tanshinone ⅡA sulfonate (STS) + Astragalus injection + Vitamin E + Coenzyme Q10; Astragalus injection + Vitamin E + Coenzyme Q10 | 58; 52 | 14 days | ECG abnormality; Response rate | S |
| Li Q, 2013 | China | 35–74 | 38 (54.3%) | Doxorubicin | Creatine phosphate sodium; No additional treatment | 35; 35 | / | ECG abnormality; FS; LVEDD; LVEF; LVESD; Number of cardiotoxicities | S |
| Li S, 2007 | China | / | 33 (73.3%) | Doxorubicin | Trimetazidine; No additional treatment | 23; 22 | 1.7 months | ECG abnormality | S |
| Li SL, 2014 | China | / | 35 (58.3%) | Doxorubicin | Ginkgo leaf extract and dipyridamole injection; No additional treatment | 30; 30 | 21 days | BNP; ECG abnormality | S |
| Li X, 2015 | China | 57.2±14.1 | 27 (44.3%) | Epirubicin | Kushen injection; No additional treatment | 30; 31 | 4.2 months | CK-MB; ECG abnormality; LDH; LVEDD; LVEF; LVESD; Number of cardiotoxicities; Troponin | S |
| Li XF, 2014 | China | 51.5±14.0 | 52 (53.1%) | Epirubicin | Trimetazidine + Shensong Yangxin capsule; No additional treatment | 49; 49 | 4.2 months | BNP; ECG abnormality; Number of cardiotoxicities; Troponin | S |
| Li XP, 2010 | China | 35.5±6.2 | 0 (0.0%) | Epirubicin | Carvedilol + Candesartan; No additional treatment | 28; 28 | 3 months | ECG abnormality; LVEDD; LVEF; LVESD; Troponin | S |
| Li YD, 2006 | China | / | 44 (55.0%) | / | Ginkgo leaf extract and dipyridamole injection; Adenosine Disodium Triphosphate + Coenzyme A | 40; 40 | / | CK; ECG abnormality; Number of cardiotoxicities | S |
| Li YQ, 2008a | China | 48.35±8.6 | 12 (33.3%) | Doxorubicin | Carvedilol; No additional treatment | 18; 18 | 6 months | 6MWD; LVEDD; LVEF; LVESD; Number of cardiotoxicities | S |
| Li YQ, 2008b | China | 38.0±8.7 | 0 (0.0%) | Doxorubicin | Astragalus injection; Polarized solution (Glucose-insulin-potassium, GIK) | 40; 40 | 4.2 months | CK-MB; E/A; ECG abnormality; LVEDD; LVEF; Number of cardiotoxicities; Troponin | S |
| Li, C., 2013 | Xiangtan, China | 53.5±9.0 | 30 (33.3%) | Doxorubicin | Astragalus injection; Shenmai injection; Dexrazoxane; | 30; 30; 30 | 3.7 months | ECG abnormality; LVEF; Troponin | S |
| Li, X., 2022 | Shanxi, China | 57.5±4.5 | 99 (44.4%) | Epirubicin | Astragalus Polysaccharide injection; No additional treatment | 113; 110 | 12 months | CK-MB; E/A; LVEF; Number of cardiotoxicities; Troponin | S |
| Liang JK, 2000 | China | 28–73 | 75 (62.5%) | Doxorubicin | SuXiaoJiuXinWan; No additional treatment | 60; 60 | / | Number of cardiotoxicities | H |
| Lin WB, 2017 | China | 46.2±5.3 | 31 (48.4%) | Epirubicin | Dexrazoxane; No additional treatment | 32; 32 | 7.5 months | BNP; Number of cardiotoxicities; Troponin | S |
| Liu JZ, 2015 | China | 0.5–16 | 48 (60.0%) | Daunorubicin | Creatine phosphate sodium; No additional treatment | 40; 40 | / | CK-MB; ECG abnormality; LVEF; Number of cardiotoxicities; Troponin | S |
| Liu L, 2013 | China | 37–68 | / | Epirubicin | Carvedilol + Candesartan; No additional treatment | 20; 20 | 4.2 months | ECG abnormality; LVEDD; LVEF; LVESD; Troponin | S |
| Liu W, 2014 | China | 4–73 | 67 (77.0%) | Doxorubicin | Vitamin C; No additional treatment | 54; 33 | 4.2 - 5.6 months | ECG abnormality; LVEF; Number of cardiotoxicities | H |
| Liu WJ, 1997 | China | 60.2±6.2 | 14 (23.3%) | / | Xinmai Long injection; No additional treatment | 29; 28 | 4.2 months | E/A; ECG abnormality; LVEDD; LVEF; LVESD; Troponin | S |
| Liu XM, 2016 | China | / | / | Pirarubicin | Dexrazoxane; No additional treatment | 25; 25 | 1.9 - 5.6 months | ECG abnormality; LVEF; Troponin | S |
| Liu Y, 2008 | China | 36–69 | 26 (32.9%) | Doxorubicin | Safflower yellow injection; Placebo | 41; 38 | / | A/E; ECG abnormality; FS; LVEDD; LVEF; LVESD | S |
| Liu YJ, 2020 | China | 44.3±4.6 | 0 (0.0%) | Epirubicin | Dexrazoxane; No additional treatment | 30; 30 | 2.8 months | CK-MB; Number of cardiotoxicities | S |
| Lu ZH, 2014 | China | 52.9±8.9 | 28 (35.0%) | Doxorubicin/  Daunorubicin | Shuxuening injection; No additional treatment | 40; 40 | 4.2 months | CK-MB; ECG abnormality; LDH; Troponin | H |
| Miao YD, 2017 | China | 55.7±10.2 | 16 (34.0%) | Doxorubicin | Calcium dibutyryladenosine cyclophosphate; No additional treatment | 24; 23 | 2.8 months | CK-MB; ECG abnormality; LVEF; NT-proBNP; Troponin | S |
| Mohamed, A. L., 2024 | Egypt | 48.8±9.9 | 0 (0.0%) | Doxorubicin/  Epirubicin | Atorvastatin; Placebo | 50; 50 | 6 months | LVEDD; LVEDV; LVEF; LVESD; LVESV; Number of cardiotoxicities | S |
| Nabati, M., 2019 | Iran | 49.3±11.3 | 0 (0.0%) | Doxorubicin | Rosuvastatin; Placebo | 39; 38 | 6 months | E/e'; GLS; LA; LVEDV; LVEF; LVESV; s';Number of cardiotoxicities | S |
| Ning YL, 2005 | China | 55.3±39.3 | 25 (80.6%) | Doxorubicin | Diosgenin; No additional treatment | 15; 16 | 2 months | ECG abnormality; LVEF; Number of cardiotoxicities | S |
| Osataphan, N., 2023 | Thailand | 53.0±11.1 | 0 (0.0%) | Doxorubicin | Metformin; Donepezil; Placebo | 41; 39; 43 | 12 months | Troponin | H |
| Qi C, 2017 | China | 38–54 | 9 (18.0%) | Doxorubicin | Shexiang Baoxin pill; No additional treatment | 25; 25 | 4.2 months | A/E; BNP; FS; LVEF; Troponin | S |
| Rahimi, K., 2023 | Iran | 7.9±4.9 | 41 (68.3%) | Doxorubicin | Desferal; No additional treatment | 20; 20; 20 | 8.0±1.8 months | AaM; AaS; AaT; AM; AT; E/AM; EaM; EaT; EDV; EM; EM/EaM; ESV; ET; FS; IVSD; IVSS; LVEDD; LVEF; LVESD; LVPWD; LVPWS; NT-proBNP; PAT; SM; SS; ST; SV | S |
| Ren HH, 2012 | China | 36–70 | 13 (17.1%) | Doxorubicin/  Epirubicin | Shenmai injection; No additional treatment | 40; 36 | 21 - 30 days | ECG abnormality; Number of cardiotoxicities | S |
| Shao Y, 2023 | China | 67.0±20.3 | 0 (0.0%) | Epirubicin | Qiliqiangxin capsule; No additional treatment | 44; 44 | 5.6 months | 6MWD; LVEDD; LVEF; LVESD; NT-proBNP | S |
| Shen N, 2010 | China | 54.1±9.0 | 91 (56.9%) | Doxorubicin | Shenfu injection; No additional treatment | 80; 80 | 4.2 months | ECG abnormality; Troponin | S |
| Slowik, A. J., 2020 | Poland | 46.3±10.5 | 0 (0.0%) | Doxorubicin/  Epirubicin | Ramipril; No additional treatment | 48; 48 | 12 months | LVEF; NT-proBNP; Number of cardiotoxicities; Troponin | S |
| Su Y, 2020 | China | 75.5±17.5 | 39 (45.3%) | Epirubicin | Dexrazoxane + Levocarnitine; Dexrazoxane | 43; 43 | 3.5 months | CK; CK-MB; ECG abnormality; LDH; Troponin | S |
| Sun CY, 2012 | China | 14–60 | 47 (56.6%) | Daunorubicin | Shenmai injection; No additional treatment | 40; 40 | 1.4 months | CK; CK-MB; NT-proBNP | S |
| Sun YS, 2012 | China | 28–65 | 32 (53.3%) | / | Coenzyme Q10; Vitamin E | 30; 30 | 1.4 months | AST; CK; ECG abnormality; HBDH; LDH; Number of cardiotoxicities | S |
| Thavendiranathan, P., 2023 | Canada | 56.9±13.7 | 87 (77.7%) | / | Atorvastatin; Placebo | 53; 55 | 2.9±1.4 months | BNP; ECV; GCS; GLS; iECV; iICV; LVEDV; LVEF; LVESV; LVM; Number of cardiotoxicities; Troponin | S |
| Wang CJ, 2003 | China | 28–77 | 0 (0.0%) | Doxorubicin | Shenmai injection + Coenzyme Q10; Coenzyme Q10 | 31; 30 | 8 days | ECG abnormality | S |
| Wang CY, 2013 | China | / | 78 (78.0%) | Doxorubicin/  Pirarubicin | Shenmai injection; No additional treatment | 50; 50 | / | AST; CK; ECG abnormality; HBDH; LDH; Number of cardiotoxicities | S |
| Wang D, 2013 | China | 0.5–17 | 45 (62.5%) | Daunorubicin | Creatine phosphate sodium; No additional treatment | 40; 32 | 1.5 - 2 months | CK-MB; ECG abnormality; LVEF; Number of cardiotoxicities; Troponin | S |
| Wang JH, 1996 | China | 25–78 | 21 (70.0%) | Doxorubicin | Vitamin C; No additional treatment | 14; 16 | / | Number of cardiotoxicities | H |
| Wang LH, 2011 | China | 13–71 | 33 (43.4%) | Daunorubicin/  Doxorubicin/  Mitoxantrone | Creatine phosphate sodium; Vitamin C | 41; 35 | 7 days | CK-MB; ECG abnormality; HBDB; LDH; Troponin | S |
| Wang QY, 2012 | China | 55.5±14.6 | 26 (42.6%) | Doxorubicin/  Epirubicin/  Pirarubicin | Shenqi Fuzheng injection; No additional treatment | 30; 31 | 4.2 months | CK-MB; ECG abnormality; LDH; LVEDD; LVEF; LVESD; Number of cardiotoxicities; Troponin | H |
| Wang SY, 2020 | China | 60–75 | 61 (50.8%) | Doxorubicin/  Epirubicin | Dexrazoxane + Shenqi Fuzheng injection; Dexrazoxane | 60; 60 | 6 months | CK; CK-MB; LVEF; Tei index; Troponin | H |
| Wang X, 2015 | China | 18–78 | / | / | Dexrazoxane; Shenmai injection; Dexrazoxane + Shenmai injection; | 35; 35; 35 | / | ECG abnormality; Troponin | S |
| Wang X, 2016 | China | 43.5±11.3 | 21 (52.5%) | / | Dexrazoxane; No additional treatment | 20; 20 | 3.5 months | ECG abnormality; LVEF; Troponin | S |
| Wang Y, 2005 | China | 18–76 | 0 (0.0%) | Doxorubicin | Zhenyuan capsule; No additional treatment | 54; 53 | 5.6 months | ECG abnormality; Number of cardiotoxicities | S |
| Wang YM, 2011 | China | 75.5±17.5 | 55 (55.0%) | Epirubicin | Levocarnitine; No additional treatment | 50; 50 | 1 days | CK; CK-MB; ECG abnormality; LDH; Number of cardiotoxicities | S |
| Wen CH, 2009 | China | 25–72 | 18 (20.5%) | Doxorubicin/  Epirubicin/  Pirarubicin | Coix Seed Oil; No additional treatment | 44; 44 | 2.1 months | E/A; FS; LVEF; Number of cardiotoxicities | S |
| Wihandono, A., 2021 | Indonesia | 47.6±8.2 | 0 (0.0%) | Doxorubicin | Lisinopril + Bisoprolol; No additional treatment | 26; 25 | 4.2 months | LVEF | S |
| Wu BR, 2015 | China | 29–72 | 12 (20.0%) | Epirubicin | Dexrazoxane; No additional treatment | 32; 28 | 6 months | BNP; ECG abnormality; Troponin | S |
| Wu SN, 2023 | China | 55.8±16.9 | 0 (0.0%) | Epirubicin | Enalapril; No additional treatment | 134; 133 | 4.7±2.6 years | ECG abnormality; GLS; LVEDD; LVEF; LVESD; NT-proBNP; Number of cardiotoxicities | S |
| Xu S, 2023 | China | 50.4±2.2 | 29 (43.9%) | / | Wenxin grannle; Placebo | 33; 33 | 2 months | Response rate | S |
| Xu X, 2016 | China | 60.2±6.2 | 14 (23.3%) | / | Xinmai Long injection; No additional treatment | 29; 28 | 4.2 months | E/A; LVEDD; LVEF; LVESD; Troponin | S |
| Xu ZL, 2008 | China | 35–67 | 0 (0.0%) | Epirubicin | Shenfu injection; No additional treatment | 27; 27 | 1.4 months | ECG abnormality; Number of cardiotoxicities | S |
| Xue XP, 2011 | China | 60–73 | 19 (45.2%) | Pirarubicin | Shenmai injection; No additional treatment | 22; 20 | 1.4 months | ECG abnormality | S |
| Yan ZM, 2021 | China | 52.9±7.1 | 0 (0.0%) | Doxorubicin | Sacubitril/Valsartan sodium; No additional treatment | 35; 35 | 3 months | LVEDD; LVEF; Response rate | S |
| Yang J, 2016 | China | 55.5±15.0 | 26 (42.6%) | / | Astragalus injection; No additional treatment | 30; 31 | 4.2 months | ECG abnormality; LVEDD; LVEF; LVESD; Number of cardiotoxicities | S |
| Yang J, 2018 | China | 50.4±3.4 | 0 (0.0%) | Epirubicin | Carvedilol + Candesartan; No additional treatment | 55; 55 | 4.2 months | ECG abnormality; LVEDD; LVEF; LVESD; Troponin | S |
| Yang JQ, 2010 | China | 51.7±7.6 | 0 (0.0%) | Epirubicin | Trimetazidine hydrochloride + Carvedilol; No additional treatment | 50; 50 | 4.2 months | ECG abnormality; LVEDD; LVEF; Troponin | S |
| Yang MD, 2022 | China | 46.9±8.5 | 0 (0.0%) | Epirubicin | Dexrazoxane; No additional treatment | 45; 45 | 5.6 months | AST; CK; CK-MB; ECG abnormality; HBDH;LDH; LVEF | S |
| Yang SL, 2019 | China | 31.8±4.3 | 53 (53.0%) | Daunorubicin | Xinmai Long injection; No additional treatment | 50; 50 | 2.1 months | CK; E/A; ECG abnormality; HBDH; LDH; LVEDD; LVEF; LVESD; NT-proBNP; Number of cardiotoxicities; Troponin | S |
| Yang XL, 2008 | China | 47.2±6.3 | 22 (37.3%) | Doxorubicin | Shengmai injection; Coenzyme Q10 + Vitamin E | 30; 29 | / | ECG abnormality; FS; LVEDD; LVEF; LVESD; Number of cardiotoxicities | S |
| Yu MJ, 2022 | China | 50.9±5.9 | 0 (0.0%) | Epirubicin | Dexrazoxane; No additional treatment | 50; 50 | 2.8 months | ECG abnormality; LVEF; Number of cardiotoxicities; Troponin | S |
| Zhai ZW, 2007 | China | 20–79 | 63 (52.5%) | Doxorubicin | Metoprolol + Polarized solution (Glucose-insulin-potassium, GIK) + Potassium aspartate and magnesium aspartate injection; No additional treatment | 60; 60 | 6 months | ECG abnormality; Number of cardiotoxicities | S |
| Zhang GW, 2016 | China | 47.9±5.1 | 91 (54.8%) | Doxorubicin | Dexrazoxane; Dexrazoxane + Cinobufacini injection; No additional treatment | 61; 75; 30 | 2.8 months | A/E; CK; CK-MB; ECG abnormality; FS; LVEDD; LVEF; LVESD; Number of cardiotoxicities | S |
| Zhang HB, 2012 | China | l6–62 | 87 (54.4%) | Doxorubicin | Zhenqi Fuzheng granules; No additional treatment | 80; 80 | 2.8 - 4.2 months | A/E; CK-MB; ECG abnormality; FS; LVEDD; LVEF; LVESD; Troponin | H |
| Zhang JP, 2009 | China | 45.2±6.1 | 29 (48.3%) | Doxorubicin | Astragalus injection; Coenzyme Q10 + Vitamin E | 30; 30 | / | A/E; CK; CK-MB; ECG abnormality; FS; LVEDD; LVEF; LVESD | S |
| Zhang LQ, 2015 | China | 4.7±1.7 | 33 (55.0%) | / | Levocarnitine; Vitamin C + Coenzyme Q10 + Vitamin E | 30; 30 | / | CK; Response rate | S |
| Zhang XJ, 2019 | China | 0.83–14 | 36 (60.0%) | Doxorubicin | Creatine phosphate sodium; No additional treatment | 30; 30 | / | ECG abnormality; Number of cardiotoxicities | S |
| Zhang YC, 2020 | China | 50.9±14.4 | 11 (27.5%) | Doxorubicin | Shengmai injection; Vitamin C + Coenzyme Q10 + Vitamin E | 20; 20 | 7 days | Number of cardiotoxicities; Troponin | S |
| Zhang YC, 2022 | China | / | / | Daunorubicin | Dexrazoxane; Vitamin C | 21; 22 | 3 months | ECG abnormality; LVEF; NT-proBNP; Number of cardiotoxicities; Troponin | S |
| Zhang YK, 2007 | China | 49.9±10.3 | 0 (0.0%) | / | Saffron total glycoside tablet; Placebo | 38; 37 | 6 months | CK-MB; ECG abnormality; LVEF; NT-proBNP; Troponin | S |
| Zhao BF, 2017 | China | 44.1±10.7 | 18 (60.0%) | Epirubicin | Dexrazoxane; No additional treatment | 15; 15 | 2.8 months | ECG abnormality; LVEF; Troponin | S |
| Zhao F, 2015 | China | 18.3–79 | 34 (42.5%) | Pirarubicin | Dexrazoxane; Polarized solution (Glucose-insulin-potassium, GIK) | 40; 40 | / | ECG abnormality | S |
| Zhao L, 2019 | China | 49.0±9.1 | 0 (0.0%) | Doxorubicin | Dexrazoxane; No additional treatment | 58; 58 | 4.2 months | BNP; CK-MB; LVEF; Troponin | S |
| Zhong L, 2017 | China | 44.7±7.5 | 0 (0.0%) | Epirubicin | Dexrazoxane; No additional treatment | 34; 34 | 1.9 months | BNP; ECG abnormality; LVEF; Troponin | S |
| Zhou AM, 2011 | China | 31–74 | 39 (43.3%) | / | Amiodarone; Wenxin grannle | 45; 45 | 2 months | Response rate | S |
| Zhou C, 2017 | China | 39–79 | 0 (0.0%) | Doxorubicin | Compound salvia miltiorrhiza injection + Levocarnitine; No additional treatment | 33; 32 | 2.1 - 4.2 months | ECG abnormality; LVEF; Troponin | S |
| Zhou GH, 2005 | China | 45.2±6.1 | 29 (50.0%) | Doxorubicin | Astragalus injection; Coenzyme Q10 + Vitamin E | 30; 28 | 7 days | A/E; ECG abnormality; FS; LVEDD; LVEF; LVESD; Number of cardiotoxicities | S |
| Zhou Y, 2021 | China | 48.0±7.4 | 0 (0.0%) | / | Levocarnitine; No additional treatment | 46; 46 | 10.2 months | LVEF | H |
| Zhuo XJ, 2018 | China | 56.1±7.8 | 36 (60.0%) | Epirubicin/  Pirarubicin | Danshen injection; No additional treatment | 30; 30 | 4.2 months | CK-MB; E/A; LVEF; Number of cardiotoxicities; Tei index; Troponin | S |
| Zou Y, 2017 | China | 42.5±9.6 | 14 (21.9%) | / | Xinmai Long injection; No additional treatment | 31; 30 | 1.5 months | 6MWD; E/A; ECG abnormality; LVEDD; LVEF; Troponin | S |
