# Supplementary material for: Prevention and treatment of anthracycline-induced cardiotoxicity: a systematic review and network meta-analysis of randomized controlled trials
Source: Cardiooncology. 2025 Jul 10;11:66. doi: 10.1186/s40959-025-00360-3 (PMC12243438; doi:10.1186/s40959-025-00360-3)
Supplement: Supplementary file 3 — Supplementary Material 3. [file 40959_2025_360_MOESM3_ESM.docx]

Prevention and Treatment of Anthracycline-Induced Cardiotoxicity: A Systematic Review and Network Meta-analysis of Randomized Controlled Trials

Current Oncology Reports

Siyu Li, MD^a¶^, Wenrui Li, MD ^a¶^, Mengfei Cheng, MD ^a^, Xiaoxiao Wang, PhD ^a^, [Wanyi Chen](http://www.frontiersin.org/Community/WhosWhoActivity.aspx?sname=WanyiChen&UID=2784852), PhD ^a^*

Affiliations

1. Department of Pharmacy, Chongqing University Cancer Hospital, Chongqing, China.

¶These authors contributed equally to this work.

* Corresponding author

E-mail: [chenwanyi@cqu.edu.cn](mailto:chenwanyi@cqu.edu.cn) (WCh)

Present address: No. 181, Hanyu Road, Shapingba District, Chongqing, China.

Table 1 Risk of bias in studies

| Study id | Risk of bias (Low/High/Some concerns) | | | | | |
| --- | --- | --- | --- | --- | --- | --- |
|  | Bias arising from  the randomisation process | Bias due to deviations from  intended interventions | Bias due to  missing outcome data | Blinding of  outcome assessment | Bias in selection of  the reported result | Overall bias |
| Abuosa, A. M., 2018 | L | L | L | L | L | L |
| Acar, Z., 2011 | L | S | L | L | L | S |
| Armenian, S. H., 2024 | L | S | L | L | L | L |
| Attar, A., 2022 | L | L | L | L | L | L |
| Avila, M. S., 2018 | L | L | L | L | L | L |
| Cai XZ, 2013 | S | S | L | S | L | S |
| Chen BB, 2016 | L | S | L | S | L | S |
| Chen CM, 2020 | L | S | L | S | L | S |
| Chen HL, 2009 | S | S | L | S | L | S |
| Chen JG, 2009 | S | S | L | S | L | S |
| Chen JT, 2015 | L | S | L | S | L | S |
| Chen R, 2012 | H | S | L | S | L | H |
| Chen RY, 2006 | S | S | L | S | L | S |
| Chen ZS, 1998 | S | S | L | S | L | S |
| Chen ZY, 2022 | L | S | L | S | L | S |
| Ciburiene, E., 2023 | L | S | L | L | L | S |
| Cochera, F., 2018 | L | S | L | L | L | S |
| Cui YZ, 2011 | S | S | L | S | L | S |
| Davis, M. K., 2019 | L | L | L | L | L | L |
| Dessi, M., 2011 | L | L | L | S | L | S |
| Dong HP, 2011 | S | S | L | S | L | S |
| Dong JH, 2007 | S | S | L | S | L | S |
| Fan LD, 2016 | L | S | L | S | L | S |
| Georgakopoulos, P., 2010 | L | S | H | L | L | H |
| Gong YL, 2008 | S | S | L | S | L | S |
| Gu B, 2011 | S | S | L | S | L | S |
| Gulati, G., 2016 | L | L | L | L | L | L |
| Hao W, 2007 | S | S | L | S | L | S |
| Hao W, 2016 | L | L | L | L | L | L |
| Hao, W., 2020 | L | L | L | L | L | L |
| He JC, 2016 | S | S | L | S | L | S |
| He MW, 2006 | L | S | L | S | L | S |
| Henriksen, P. A., 2023 | L | H | L | L | L | H |
| Hu XB, 2004 | S | S | L | S | L | S |
| Huang LQ, 2015 | S | S | L | S | L | S |
| Hundley, W. G., 2022 | L | L | S | L | L | S |
| Jhorawat, R., 2016 | S | S | L | L | L | S |
| Jiang YQ, 2010 | S | S | L | S | L | S |
| Jiang ZH, 2018 | L | S | L | S | L | S |
| Jo, S. H., 2013 | L | S | L | L | L | S |
| Kalay, N., 2006 | S | S | L | L | L | S |
| Kong JX, 2013 | S | S | L | S | L | S |
| Kong JX, 2017 | L | S | L | S | L | S |
| Lee, M., 2021 | L | S | L | L | L | S |
| Li G, 2021 | L | S | L | S | L | S |
| Li H, 2007 | S | S | L | S | L | S |
| Li Q, 2013 | S | S | L | S | L | S |
| Li S, 2007 | S | S | L | S | L | S |
| Li SL, 2014 | S | S | L | S | L | S |
| Li X, 2015 | S | S | L | S | L | S |
| Li XF, 2014 | L | S | L | S | L | S |
| Li XP, 2010 | S | S | L | S | L | S |
| Li YD, 2006 | S | S | L | S | S | S |
| Li YQ, 2008a | S | S | L | S | S | S |
| Li YQ, 2008b | S | S | L | S | L | S |
| Li, C., 2013 | S | S | L | S | L | S |
| Li, X., 2022 | S | S | L | S | L | S |
| Liang JK, 2000 | H | S | L | S | L | H |
| Lin WB, 2017 | S | S | L | S | L | S |
| Liu JZ, 2015 | L | S | L | S | L | S |
| Liu L, 2013 | S | S | L | S | L | S |
| Liu W, 2014 | H | S | L | S | L | H |
| Liu WJ, 1997 | L | S | L | S | L | S |
| Liu XM, 2016 | S | S | L | S | L | S |
| Liu Y, 2008 | S | S | L | S | L | S |
| Liu YJ, 2020 | S | S | L | S | L | S |
| Lu ZH, 2014 | H | S | L | S | L | H |
| Miao YD, 2017 | L | S | L | S | L | S |
| Mohamed, A. L., 2024 | L | S | L | L | L | S |
| Nabati, M., 2019 | L | S | L | L | L | S |
| Ning YL, 2005 | S | S | L | S | L | S |
| Osataphan, N., 2023 | H | L | L | L | L | H |
| Qi C, 2017 | L | S | L | S | L | S |
| Rahimi, K., 2023 | L | S | L | L | L | S |
| Ren HH, 2012 | S | S | L | S | L | S |
| Shao Y, 2023 | S | S | L | S | L | S |
| Shen N, 2010 | L | S | L | S | L | S |
| Slowik, A. J., 2020 | L | S | L | L | L | S |
| Su Y, 2020 | L | S | L | S | L | S |
| Sun CY, 2012 | L | S | L | S | L | S |
| Sun YS, 2012 | S | S | L | S | L | S |
| Thavendiranathan, P., 2023 | S | L | L | S | L | S |
| Wang CJ, 2003 | S | S | L | S | L | S |
| Wang CY, 2013 | S | S | L | S | S | S |
| Wang D, 2013 | S | S | L | S | L | S |
| Wang JH, 1996 | H | S | L | S | L | H |
| Wang LH, 2011 | S | S | L | S | L | S |
| Wang QY, 2012 | H | S | L | S | L | H |
| Wang SY, 2020 | H | S | L | S | L | H |
| Wang X, 2015 | S | S | L | S | L | S |
| Wang X, 2016 | S | S | L | S | L | S |
| Wang Y, 2005 | S | S | L | S | S | S |
| Wang YM, 2011 | S | S | L | S | L | S |
| Wen CH, 2009 | S | S | L | S | L | S |
| Wihandono, A., 2021 | S | L | S | S | L | S |
| Wu BR, 2015 | S | S | L | S | L | S |
| Wu SN, 2023 | L | S | L | S | L | S |
| Xu S, 2023 | L | S | L | S | L | S |
| Xu X, 2016 | L | S | L | S | L | S |
| Xu ZL, 2008 | S | S | L | S | L | S |
| Xue XP, 2011 | S | S | L | S | L | S |
| Yan ZM, 2021 | S | S | L | S | L | S |
| Yang J, 2016 | S | S | L | S | L | S |
| Yang J, 2018 | S | S | L | S | L | S |
| Yang JQ, 2010 | L | S | L | S | L | S |
| Yang MD, 2022 | S | S | L | S | L | S |
| Yang SL, 2019 | S | S | L | S | L | S |
| Yang XL, 2008 | S | S | L | S | L | S |
| Yu MJ, 2022 | L | S | L | S | L | S |
| Zhai ZW, 2007 | S | S | L | S | L | S |
| Zhang GW, 2016 | S | S | L | S | L | S |
| Zhang HB, 2012 | H | S | L | S | L | H |
| Zhang JP, 2009 | S | S | L | S | L | S |
| Zhang LQ, 2015 | S | S | L | S | L | S |
| Zhang XJ, 2019 | L | S | L | S | L | S |
| Zhang YC, 2020 | L | S | L | S | L | S |
| Zhang YC, 2022 | S | S | L | S | L | S |
| Zhang YK, 2007 | L | L | L | S | L | S |
| Zhao BF, 2017 | S | S | L | S | L | S |
| Zhao F, 2015 | S | S | L | S | S | S |
| Zhao L, 2019 | L | S | L | S | L | S |
| Zhong L, 2017 | S | S | L | S | L | S |
| Zhou AM, 2011 | S | S | L | S | L | S |
| Zhou C, 2017 | S | S | L | S | L | S |
| Zhou GH, 2005 | S | S | L | S | L | S |
| Zhou Y, 2021 | H | S | L | S | L | H |
| Zhuo XJ, 2018 | L | S | L | S | L | S |
| Zou Y, 2017 | S | S | L | S | L | S |

Note: L: Low risk of bias; S: Some concerns; H: High risk of bias.
