# Supplementary material for: Prevention and treatment of anthracycline-induced cardiotoxicity: a systematic review and network meta-analysis of randomized controlled trials
Source: Cardiooncology. 2025 Jul 10;11:66. doi: 10.1186/s40959-025-00360-3 (PMC12243438; doi:10.1186/s40959-025-00360-3)
Supplement: Supplementary file 4 — Supplementary Material 4. [file 40959_2025_360_MOESM4_ESM.docx]

Prevention and Treatment of Anthracycline-Induced Cardiotoxicity: A Systematic Review and Network Meta-analysis of Randomized Controlled Trials

Current Oncology Reports

Siyu Li, MD^a¶^, Wenrui Li, MD ^a¶^, Mengfei Cheng, MD ^a^, Xiaoxiao Wang, PhD ^a^, [Wanyi Chen](http://www.frontiersin.org/Community/WhosWhoActivity.aspx?sname=WanyiChen&UID=2784852), PhD ^a^*

Affiliations

1. Department of Pharmacy, Chongqing University Cancer Hospital, Chongqing, China.

¶These authors contributed equally to this work.

* Corresponding author

E-mail: [chenwanyi@cqu.edu.cn](mailto:chenwanyi@cqu.edu.cn) (WCh)

Present address: No. 181, Hanyu Road, Shapingba District, Chongqing, China.

- Node-splitting analysis of inconsistency

LVEF

| Comparison | P-value | Mean Difference (95% CrI) |
| --- | --- | --- |
| Astragalus polysaccharide vs **Creatine phosphate sodium** | 0.8155 |  |
|  | direct | -2.6 (-8.4, 3.1) |
|  | indirect | -0.47 ( -18., 17.) |
|  | network | -2.4 (-7.8, 3.) |
| Candesartan vs **Carvedilol** | 0.48706 |  |
|  | direct | -1.2 (-9.4, 7.) |
|  | indirect | 2.8 (-5.5, 11.) |
|  | network | 0.71 (-5.0, 6.5) |
| Candesartan vs **Metoprolol** | 0.50598 |  |
|  | direct | -1.6 (-9.9, 6.7) |
|  | indirect | -6.4 ( -19., 5.8) |
|  | network | -3.1 (-9.7, 3.5) |
| Astragalus vs **DEX** | 0.71579 |  |
|  | direct | 0.068 (-7.9, 8.0) |
|  | indirect | 3.2 ( -12., 18.) |
|  | network | 0.71 (-6.2, 7.7) |
| Enalapril vs **Metoprolol** | 0.98313 |  |
|  | direct | -1.1 (-9.7, 7.6) |
|  | indirect | -1.2 ( -13., 10.) |
|  | network | -1.1 (-7.8, 5.5) |
| Astragalus polysaccharide vs **Placebo/No additional treatment** | 0.82197 |  |
|  | direct | -4.2 (-12., 4.) |
|  | indirect | -6.3 ( -23., 10.) |
|  | network | -4.6 ( -12., 2.6) |
| Astragalus vs **Placebo/No additional treatment** | 0.71783 |  |
|  | direct | -2.0 (-16., 12.) |
|  | indirect | -5.1 (-13., 3.3) |
|  | network | -4.4 (-11., 2.8) |
| Candesartan vs **Placebo/No additional treatment** | 0.5343 |  |
|  | direct | -1.9 (-10., 6.5) |
|  | indirect | -5.7 ( -15., 3.4) |
|  | network | -3.3 (-9.0, 2.4) |
| Carvedilol vs **Placebo/No additional treatment** | 0.48852 |  |
|  | direct | -4.5 (-8.4, -0.73) |
|  | indirect | -0.48 ( -12., 11.) |
|  | network | -4.0 (-7.6, -0.55) |
| Creatine phosphate sodium vs **Placebo/No additional treatment** | 0.81021 |  |
|  | direct | -3.8 (-19., 12.) |
|  | indirect | -1.5 ( -12., 8.5) |
|  | network | -2.2 ( -11., 6.0) |
| DEX vs **Placebo/No additional treatment** | 0.72484 |  |
|  | direct | -5.1 (-7.7, -2.6) |
|  | indirect | -2.2 ( -19., 14.) |
|  | network | -5.1 (-7.5, -2.6) |

Abnormal ECG

| Comparison | P-value | Odds Ratio (95% CrI) |
| --- | --- | --- |
| Astragalus vs Coenzyme Q10+Vitamin E | 0.76671 |  |
|  | direct | 4.384 (1.554,13.44) |
|  | indirect | 5.900 (1.088,34.31) |
|  | network | 4.682 (1.952,11.78) |
| Astragalus vs Shenmai | 0.48817 |  |
|  | direct | 0.8495 (0.1918,3.677) |
|  | indirect | 1.610 (0.5205,5.101) |
|  | network | 1.250 (0.5335,2.944) |
| Astragalus vs DEX | 0.64065 |  |
|  | direct | 0.8277 (0.1940,3.533) |
|  | indirect | 1.243 (0.4164,3.878) |
|  | network | 1.013 (0.4534,2.443) |
| DEX vs GIK | 0.11257 |  |
|  | direct | 5.557 (1.491,24.37) |
|  | indirect | 34.45 (6.179,239.2) |
|  | network | 11.29 (3.739,37.60) |
| DEX vs Shenmai | 0.74904 |  |
|  | direct | 1.361 (0.4911,3.859) |
|  | indirect | 1.092 (0.3784,2.926) |
|  | network | 1.222 (0.5959,2.400) |
| DEX vs Vitamin C | 0.20032 |  |
|  | direct | 3.188 (0.5630,19.96) |
|  | indirect | 0.4435 (0.01356,4.811) |
|  | network | 1.627 (0.4137,6.231) |
| Astragalus vs Placebo/No additional treatment | 0.85082 |  |
|  | direct | 4.422 (1.024,21.05) |
|  | indirect | 5.293 (1.828,15.04) |
|  | network | 4.931 (2.105,11.61) |
| DEX vs Placebo/No additional treatment | 0.83913 |  |
|  | direct | 4.633 (2.438,8.687) |
|  | indirect | 5.288 (1.876,15.37) |
|  | network | 4.805 (2.803,8.184) |
| Enalapril+Metoprolol vs Placebo/No additional treatment | 0.49934 |  |
|  | direct | 14.66 (3.695,62.99) |
|  | indirect | 6.252 (0.7893,60.32) |
|  | network | 10.61 (3.464,35.33) |
| Placebo/No additional treatment vs Shenmai | 0.86244 |  |
|  | direct | 0.2644 (0.1083,0.6258) |
|  | indirect | 0.2352 (0.08748,0.6619) |
|  | network | 0.2534 (0.1315,0.4868) |
| Placebo/No additional treatment vs Vitamin C | 0.20131 |  |
|  | direct | 0.1013 (0.003292,1.007) |
|  | indirect | 0.6753 (0.1148,5.264) |
|  | network | 0.3414 (0.08390,1.305) |
| Astragalus vs GIK | 0.10124 |  |
|  | direct | 26.69 (6.107,150.8) |
|  | indirect | 4.144 (0.8560,24.61) |
|  | network | 11.63 (3.903,38.23) |
| Coenzyme Q10+Vitamin E vs Shenmai | 0.77434 |  |
|  | direct | 0.2303 (0.05477,0.8873) |
|  | indirect | 0.3054 (0.06917,1.293) |
|  | network | 0.2652 (0.09757,0.7013) |

Symptomatic cardiotoxicity

| Comparison | P-value | Odds Ratio (95% CrI) |
| --- | --- | --- |
| Astragalus vs Coenzyme Q10+Vitamin E | 0.92534 |  |
|  | direct | 6.995 (0.5178,300.1) |
|  | indirect | 5.919 (0.1984,172.5) |
|  | network | 5.765 (0.8838,48.09) |
| Astragalus polysaccharide vs Creatine phosphate sodium | 0.54967 |  |
|  | direct | 1.586 (0.2963,8.690) |
|  | indirect | 3.930 (0.2042,57.12) |
|  | network | 1.967 (0.5333,7.353) |
| Enalapril vs Metoprolol | 0.50225 |  |
|  | direct | 0.4024 (0.009469,7.388) |
|  | indirect | 9.983e-12 (1.593e-44,7.892e+16) |
|  | network | 0.3390 (0.01136,3.622) |
| Astragalus vs Placebo/No additional treatment | 0.94253 |  |
|  | direct | 3.813 (0.4926,42.20) |
|  | indirect | 4.470 (0.1423,352.8) |
|  | network | 3.723 (0.6702,26.53) |
| Astragalus polysaccharide vs Placebo/No additional treatment | 0.53599 |  |
|  | direct | 8.744 (1.886,42.80) |
|  | indirect | 3.577 (0.2307,72.90) |
|  | network | 7.493 (2.246,24.41) |
| Creatine phosphate sodium vs Placebo/No additional treatment | 0.54012 |  |
|  | direct | 2.301 (0.2649,24.39) |
|  | indirect | 5.553 (0.6269,52.73) |
|  | network | 3.843 (0.8635,16.88) |
| Shenmai vs Placebo/No additional treatment | 0.96184 |  |
|  | direct | 0.3090 (0.04571,1.428) |
|  | indirect | 0.3293 (0.006646,28.35) |
|  | network | 0.3178 (0.06694,1.236) |
| Coenzyme Q10+Vitamin E vs Shenmai | 0.94901 |  |
|  | direct | 0.2042 (0.03661,1.029) |
|  | indirect | 0.1741 (0.001527,8.421) |
|  | network | 0.2066 (0.04500,0.7941) |

- Analysis of heterogeneity

LVEF

| Comparison | I^2^.pair | I^2^.cons |
| --- | --- | --- |
| Shenqi Fuzheng vs Placebo/No additional treatment | 71.61764 | 71.54736 |
| Xinmai Long vs Placebo/No additional treatment | 55.74267 | 55.769 |
| Candesartan vs Placebo/No additional treatment | NA | 10.3186 |
| Enalapril vs Placebo/No additional treatment | 62.51767 | 53.82613 |
| Metoprolol vs Placebo/No additional treatment | 0 | 0 |
| Saffron total glycoside vs Placebo/No additional treatment | 58.39488 | 57.36974 |
| Calcium Dibutyryladenosine Cyclophosphate vs Placebo/No additional treatment | NA | NA |
| Candesartan+Metoprolol vs Placebo/No additional treatment | NA | NA |
| DEX vs Placebo/No additional treatment | 73.41906 | 70.6532 |
| Coix Seed Oil vs Placebo/No additional treatment | NA | NA |
| Compound salvia miltiorrhiza+Levocarnitine vs Placebo/No additional treatment | NA | NA |
| Danshen vs Placebo/No additional treatment | NA | NA |
| DEX+Cinobufacini vs Placebo/No additional treatment | NA | NA |
| Diosgenin vs Placebo/No additional treatment | NA | NA |
| Eplerenone vs Placebo/No additional treatment | NA | NA |
| Ivabradine vs Placebo/No additional treatment | NA | NA |
| Kushen vs Placebo/No additional treatment | NA | NA |
| Levocarnitine vs Placebo/No additional treatment | NA | NA |
| Carvedilol vs Placebo/No additional treatment | 95.93671 | 94.33072 |
| Lisinopril+Bisoprolol vs Placebo/No additional treatment | NA | NA |
| N-acetylcysteine vs Placebo/No additional treatment | NA | NA |
| Nebivolol vs Placebo/No additional treatment | NA | NA |
| Nicorandil vs Placebo/No additional treatment | NA | NA |
| Platycodon grandiflorum vs Placebo/No additional treatment | NA | NA |
| Qiliqiangxin vs Placebo/No additional treatment | NA | NA |
| GSH vs Placebo/No additional treatment | NA | NA |
| Rosuvastatin vs Placebo/No additional treatment | NA | NA |
| Astragalus vs Placebo/No additional treatment | NA | 0 |
| Safflower yellow vs Placebo/No additional treatment | NA | NA |
| Shexiang Baoxin vs Placebo/No additional treatment | NA | NA |
| Sildenafil vs Placebo/No additional treatment | NA | NA |
| Telmisartan vs Placebo/No additional treatment | NA | NA |
| Trimetazidine+Carvedilol vs Placebo/No additional treatment | NA | NA |
| Yiqi Fumai vs Placebo/No additional treatment | NA | NA |
| Atorvastatin vs Placebo/No additional treatment | 99.73663 | 99.73337 |
| Astragalus polysaccharide vs Placebo/No additional treatment | NA | 0 |
| Carvedilol+Candesartan vs Placebo/No additional treatment | 99.71558 | 99.71229 |
| Creatine phosphate sodium vs Placebo/No additional treatment | NA | 0 |
| Metoprolol vs Candesartan | NA | 29.5548 |
| Candesartan+Metoprolol vs Candesartan | NA | NA |
| Carvedilol vs Candesartan | NA | 75.03337 |
| Metoprolol vs Enalapril | NA | 0 |
| Candesartan+Metoprolol vs Metoprolol | NA | NA |
| DEX vs Astragalus+DEX | NA | NA |
| DEX+Cinobufacini vs DEX | NA | NA |
| DEX+Shenqi Fuzheng vs DEX | NA | NA |
| Astragalus vs DEX | NA | 0 |
| Shenmai vs DEX | NA | NA |
| Vitamin C vs DEX | NA | NA |
| Astragalus vs GIK | NA | NA |
| Coenzyme Q10+Vitamin E vs Safflower | NA | NA |
| Shenmai vs Astragalus | NA | NA |
| Coenzyme Q10+Vitamin E vs Astragalus | 0 | 0 |
| Coenzyme Q10+Vitamin E vs Shengmai | NA | NA |
| Creatine phosphate sodium vs Astragalus polysaccharide | 91.27818 | 81.7394 |
| Global I-squared | 98.60506 | 98.1336 |

Abnormal ECG

| Comparison | I^2^.pair | I^2^.cons |
| --- | --- | --- |
| Shenqi Fuzheng vs Placebo/No additional treatment | 39.98605 | 39.862522 |
| Xinmai Long vs Placebo/No additional treatment | 10.05803 | 9.67373 |
| Enalapril vs Placebo/No additional treatment | 22.43481 | 29.467736 |
| Metoprolol vs Placebo/No additional treatment | NA | NA |
| Saffron total glycoside vs Placebo/No additional treatment | 0 | 0 |
| Calcium Dibutyryladenosine Cyclophosphate vs Placebo/No additional treatment | NA | NA |
| DEX vs Placebo/No additional treatment | 22.12579 | 10.648502 |
| Compound salvia miltiorrhiza+Levocarnitine vs Placebo/No additional treatment | NA | NA |
| DEX+Cinobufacini vs Placebo/No additional treatment | NA | NA |
| Diosgenin vs Placebo/No additional treatment | NA | NA |
| Kushen vs Placebo/No additional treatment | NA | NA |
| Levocarnitine vs Placebo/No additional treatment | NA | NA |
| Carvedilol vs Placebo/No additional treatment | NA | NA |
| Nicorandil vs Placebo/No additional treatment | NA | NA |
| Platycodon grandiflorum vs Placebo/No additional treatment | NA | NA |
| GSH vs Placebo/No additional treatment | NA | NA |
| Astragalus vs Placebo/No additional treatment | NA | 0 |
| Safflower yellow vs Placebo/No additional treatment | NA | NA |
| Shenmai vs Placebo/No additional treatment | 0 | 0 |
| Telmisartan vs Placebo/No additional treatment | NA | NA |
| Trimetazidine+Carvedilol vs Placebo/No additional treatment | NA | NA |
| Vitamin C vs Placebo/No additional treatment | NA | 7.048742 |
| Zhenqi Fuzheng vs Placebo/No additional treatment | NA | NA |
| Shenfu vs Placebo/No additional treatment | 45.76642 | 45.732658 |
| Shuxuening vs Placebo/No additional treatment | NA | NA |
| Ginkgo Leaf Extract and Dipyridamole vs Placebo/No additional treatment | NA | NA |
| Trimetazidine+Shensong Yangxin vs Placebo/No additional treatment | NA | NA |
| Enalapril+Metoprolol vs Placebo/No additional treatment | NA | 0 |
| Potassium Aspartate and Magnesium Aspartate vs Placebo/No additional treatment | NA | NA |
| Metoprolol+GIK+Potassium Aspartate and Magnesium Aspartate vs Placebo/No additional treatment | NA | NA |
| Zhenyuan vs Placebo/No additional treatment | NA | NA |
| Carvedilol+Candesartan vs Placebo/No additional treatment | 57.45166 | 57.451003 |
| Creatine phosphate sodium vs Placebo/No additional treatment | 0 | 0 |
| Metoprolol vs Enalapril | NA | NA |
| Enalapril+Metoprolol vs Enalapril | 0 | 0 |
| Enalapril+Metoprolol vs Metoprolol | NA | NA |
| DEX vs Astragalus+DEX | NA | NA |
| DEX+Cinobufacini vs DEX | NA | NA |
| GIK vs DEX | NA | 64.734014 |
| Astragalus vs DEX | NA | 0 |
| Shenmai vs DEX | 0 | 0 |
| Vitamin C vs DEX | NA | 23.661603 |
| DEX+Levocarnitine vs DEX | NA | NA |
| DEX+Shenmai vs DEX | NA | NA |
| Astragalus vs GIK | NA | 53.43342 |
| Shenmai+Compound salvia miltiorrhiza vs GIK | NA | NA |
| Coenzyme Q10+Vitamin E vs Safflower | NA | NA |
| Shenmai vs Astragalus | NA | 0 |
| Coenzyme Q10+Vitamin E vs Astragalus | 0 | 0 |
| Vitamin C+Coenzyme Q10+Vitamin E vs Shengmai | NA | NA |
| Coenzyme Q10+Vitamin E vs Shengmai | NA | NA |
| DEX+Shenmai vs Shenmai | NA | NA |
| Coenzyme Q10+Vitamin E vs Shenmai | NA | 0 |
| Adenosine Disodium Triphosphate+Coenzyme A vs Ginkgo Leaf Extract and Dipyridamole | NA | NA |
| Global I-squared | 15.74004 | 1.536007 |

Symptomatic cardiotoxicity

| Comparison | I^2^.pair | I^2^.cons |
| --- | --- | --- |
| Shenqi Fuzheng vs Placebo/No additional treatment | NA | NA |
| Xinmai Long vs Placebo/No additional treatment | NA | NA |
| Candesartan vs Placebo/No additional treatment | NA | NA |
| Enalapril vs Placebo/No additional treatment | 0 | 0 |
| Metoprolol vs Placebo/No additional treatment | 0 | 0 |
| Saffron total glycoside vs Placebo/No additional treatment | NA | NA |
| Candesartan+Metoprolol vs Placebo/No additional treatment | NA | NA |
| DEX vs Placebo/No additional treatment | 0 | 0 |
| Coix Seed Oil vs Placebo/No additional treatment | NA | NA |
| Danshen vs Placebo/No additional treatment | NA | NA |
| DEX+Cinobufacini vs Placebo/No additional treatment | NA | NA |
| Diosgenin vs Placebo/No additional treatment | NA | NA |
| Ivabradine vs Placebo/No additional treatment | NA | NA |
| Kushen vs Placebo/No additional treatment | NA | NA |
| Levocarnitine vs Placebo/No additional treatment | NA | NA |
| Carvedilol vs Placebo/No additional treatment | 20.96693 | 21.34627 |
| Platycodon grandiflorum vs Placebo/No additional treatment | 0 | 0 |
| Rosuvastatin vs Placebo/No additional treatment | NA | NA |
| Astragalus vs Placebo/No additional treatment | NA | 0 |
| Shenmai vs Placebo/No additional treatment | 0 | 0 |
| Yixinshu vs Placebo/No additional treatment | NA | NA |
| Atorvastatin vs Placebo/No additional treatment | NA | NA |
| Shenfu vs Placebo/No additional treatment | NA | NA |
| Metoprolol+GIK+Potassium Aspartate and Magnesium Aspartate vs Placebo/No additional treatment | NA | NA |
| Zhenyuan vs Placebo/No additional treatment | NA | NA |
| SuXiaoJiuXinWan vs Placebo/No additional treatment | NA | NA |
| Astragalus polysaccharide vs Placebo/No additional treatment | NA | 0 |
| Creatine phosphate sodium vs Placebo/No additional treatment | 0 | 0 |
| Metoprolol vs Candesartan | NA | NA |
| Candesartan+Metoprolol vs Candesartan | NA | NA |
| Metoprolol vs Enalapril | NA | 0 |
| Candesartan+Metoprolol vs Metoprolol | NA | NA |
| Yixinshu vs Coenzyme Q10 | NA | NA |
| Kudiezi vs Coenzyme Q10 | NA | NA |
| Vitamin E vs Coenzyme Q10 | NA | NA |
| DEX+Cinobufacini vs DEX | NA | NA |
| Vitamin C vs DEX | NA | NA |
| Astragalus vs GIK | NA | NA |
| Shenmai+Compound salvia miltiorrhiza vs GIK | NA | NA |
| Coenzyme Q10+Vitamin E vs Astragalus | NA | 0 |
| Vitamin C+Coenzyme Q10+Vitamin E vs Shengmai | 0 | 0 |
| Coenzyme Q10+Vitamin E vs Shengmai | NA | NA |
| Coenzyme Q10+Vitamin E vs Shenmai | NA | 0 |
| Creatine phosphate sodium vs Astragalus polysaccharide | NA | 0 |
| Global I-squared | 71.79996 | 0 |

CK-MB

| Comparison | I^2^.pair | I^2^.cons |
| --- | --- | --- |
| Shenqi Fuzheng vs Placebo/No additional treatment | 95.4282 | 95.39305 |
| Saffron total glycoside vs Placebo/No additional treatment | 0 | 0 |
| Calcium Dibutyryladenosine Cyclophosphate vs Placebo/No additional treatment | NA | NA |
| DEX vs Placebo/No additional treatment | 0 | 0 |
| Danshen vs Placebo/No additional treatment | NA | NA |
| DEX+Cinobufacini vs Placebo/No additional treatment | NA | NA |
| Kushen vs Placebo/No additional treatment | NA | NA |
| Levocarnitine vs Placebo/No additional treatment | NA | NA |
| GSH vs Placebo/No additional treatment | NA | NA |
| Shenmai vs Placebo/No additional treatment | NA | NA |
| Yiqi Fumai vs Placebo/No additional treatment | NA | NA |
| Zhenqi Fuzheng vs Placebo/No additional treatment | NA | NA |
| Shenfu vs Placebo/No additional treatment | NA | NA |
| Shuxuening vs Placebo/No additional treatment | NA | NA |
| Astragalus polysaccharide vs Placebo/No additional treatment | NA | NA |
| DEX vs Astragalus+DEX | NA | NA |
| DEX+Cinobufacini vs DEX | NA | NA |
| DEX+Shenqi Fuzheng vs DEX | NA | NA |
| DEX+Levocarnitine vs DEX | NA | NA |
| Global I-squared | 83.10685 | 0 |
