# Supplementary material for: Prevention and treatment of anthracycline-induced cardiotoxicity: a systematic review and network meta-analysis of randomized controlled trials
Source: Cardiooncology. 2025 Jul 10;11:66. doi: 10.1186/s40959-025-00360-3 (PMC12243438; doi:10.1186/s40959-025-00360-3)
Supplement: Supplementary file 5 — Supplementary Material 5. [file 40959_2025_360_MOESM5_ESM.docx]

Prevention and Treatment of Anthracycline-Induced Cardiotoxicity: A Systematic Review and Network Meta-analysis of Randomized Controlled Trials

Current Oncology Reports

Siyu Li, MD^a¶^, Wenrui Li, MD ^a¶^, Mengfei Cheng, MD ^a^, Xiaoxiao Wang, PhD ^a^, [Wanyi Chen](http://www.frontiersin.org/Community/WhosWhoActivity.aspx?sname=WanyiChen&UID=2784852), PhD ^a^*

Affiliations

1. Department of Pharmacy, Chongqing University Cancer Hospital, Chongqing, China.

¶These authors contributed equally to this work.

* Corresponding author

E-mail: [chenwanyi@cqu.edu.cn](mailto:chenwanyi@cqu.edu.cn) (WCh)

Present address: No. 181, Hanyu Road, Shapingba District, Chongqing, China.


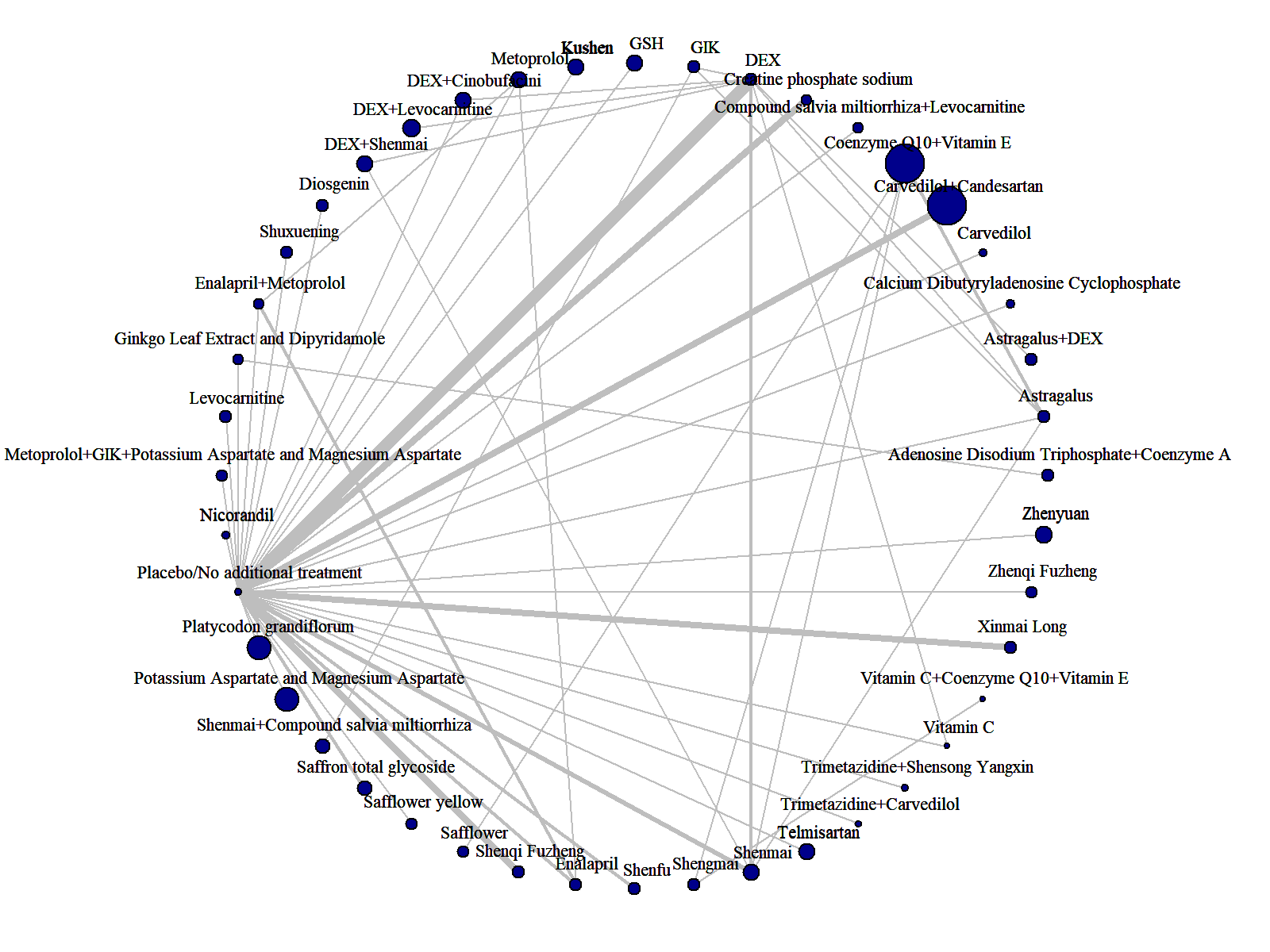


Figure 1 Network of electrocardiographic abnormality


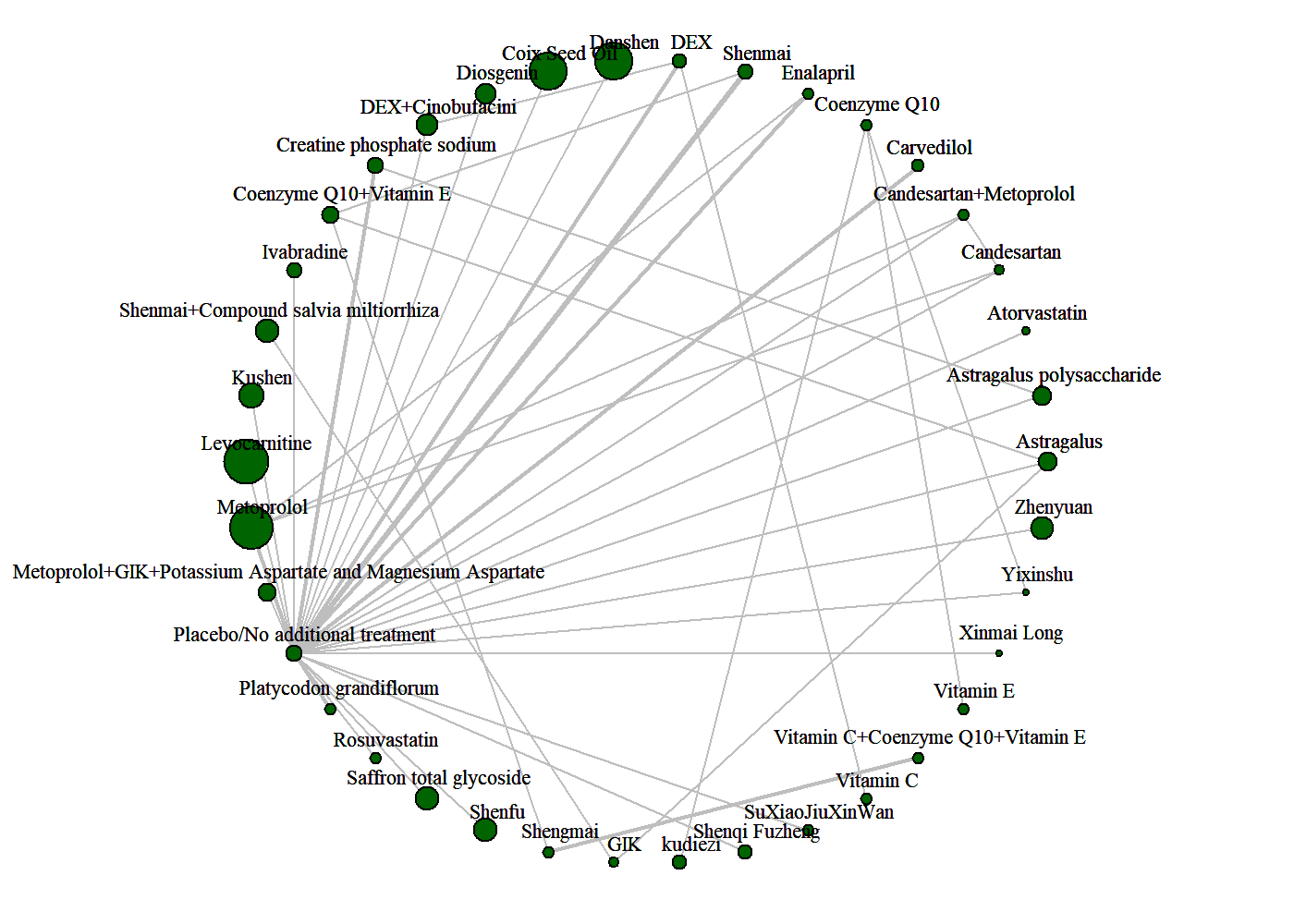


Figure 2 Network of symptomatic cardiotoxicity


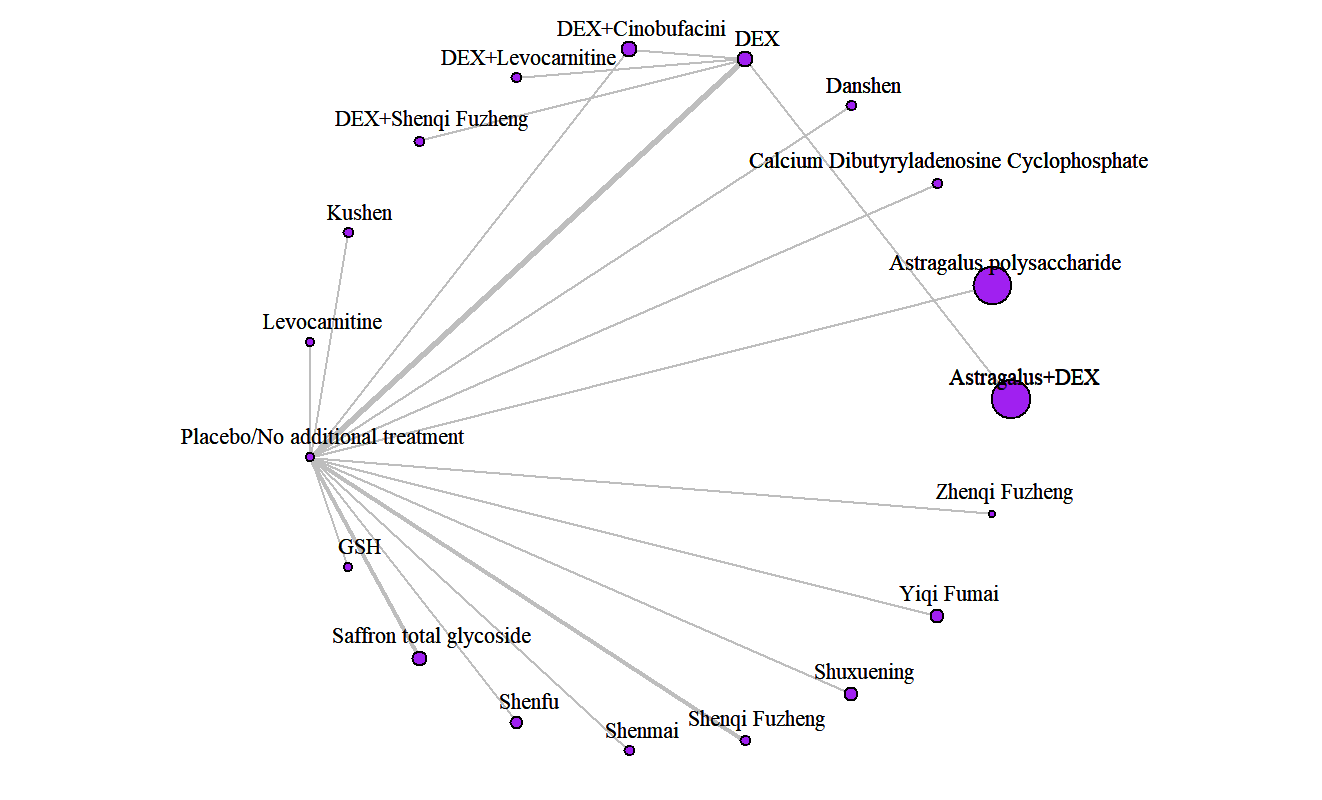


Figure 3 Network of CK-MB
